# Shared digital, social, and cognitive mechanisms in gambling and pseudo-investment schemes in Kazakhstan: a multi-source qualitative study

**DOI:** 10.3389/fpsyg.2026.1756586

**Published:** 2026-08-26

**Authors:** Zhanna Khamzina, Yermek Buribayev

**Affiliations:** Department of Law, Zhetysu University Named After I. Zhansugurov, TaldyKorgan, Kazakhstan

**Keywords:** cross-domain migration, digital, social, and cognitive mechanisms, engagement and retention trajectories, gambling-related harms, Kazakhstan, pyramidal pseudo-investment schemes (financial pyramids)

## Abstract

**Background:**

In Kazakhstan, online betting, online casinos, and pyramidal pseudo-investment schemes increasingly occupy overlapping mobile and platform-mediated environments. Shared channels may support common engagement mechanisms. Participant-level sequential entry into a second domain, continued participation in that domain, and durable migration require separate empirical assessment.

**Aim:**

Guided by three qualitative research questions, the article examines: (1) how digital, social, and cognitive mechanisms shape entry and retention; (2) which participant-level records document temporally ordered participation across gambling and pseudo-investment domains and which cross-source records document shared cross-domain infrastructure; and (3) which contextual conditions concerning recruitment, visibility, household recognition, and crisis response are illustrated in the material.

**Methods:**

A multi-source qualitative design combined 25 interviews with persons reporting gambling-related problems, 19 with pseudo-investment participants, 22 family or co-resident interviews concerning gambling-related focal persons, 22 psychologist or psychotherapist interviews, 12 law-enforcement interviews, and five macro-regional focus groups. The 44 first-person accounts formed the trajectory denominator. Respondent groups were coded within source type and integrated through provenance-preserving joint displays. Direct first-person evidence determined participant chronology and case counts; collateral and cross-source evidence informed mechanism interpretation. Cross-domain coding used three grades: temporally ordered second-domain entry, continued participation beyond a single trial, and durable substitution or reorientation. Direction-specific elicitation was systematic for P → G in the pseudo-investment guide and general for G → P in the gambling guide.

**Results:**

Case-level mapping identified five recurrent within-domain stages and unevenly supported adjacent links. Eleven cases documented the complete five-stage order, 17 contained other partial ordered sequences, 12 were nonlinear, recursive, or alternative, and 4 were trial-only or nonconsolidating. Adjacent transitions were EXP → ENT in 35 cases, ENT → CON in 39, CON→ESC in 30, and ESC → CRS in 27. Thirteen unique cases bypassed at least one intermediate stage. The tested six-element order with cross-domain change between escalation and crisis/exit occurred in zero cases. Two of the 19 pseudo-investment participants with systematic adjacent-risk elicitation documented post-crisis P → G entry; these were also two of the 44 first-person cases overall. P04 progressed to repeated gambling and escalation. P17 reported one lottery purchase. The durable-migration criterion was unmet in both cases. Direct records contained zero G → P cases under general elicitation. Regional metadata were reportable for 20 of 44 first-person cases and unavailable or suppressed for 24. The contextual analysis centered on mechanisms, with region and gender retained as source metadata.

**Conclusion:**

The data support a heterogeneous cross-case framework of recurrent within-domain stages and uneven transition possibilities. Cross-domain evidence comprised two post-crisis P → G entries: one case progressed to repeated second-domain participation, and one remained a single trial. Durable cross-domain migration occurred in zero cases. The study integrates three evidence categories: recurrent shared mechanisms, shared cross-domain infrastructure, and graded participant-level sequential entry.

## 1 Introduction

Online gambling, online casinos, sports betting, and pyramidal pseudo-investment schemes increasingly reach participants through the same mobile and platform-mediated environments. Advertising encounters, social-media feeds, messenger invitations, influencer endorsements, rapid payments, and short-term credit may form a connected infrastructure through which visibility, recruitment, trial participation, repeated payments, and attempts at recovery are organized across adjacent digital products. The analysis examines whether this infrastructure appears within individual participant trajectories. It addresses recurrent mechanisms and documented case-level movement in Kazakhstan, where gambling and pyramidal pseudo-investment schemes have distinct legal and commercial status.

Kazakhstan provides a relevant setting for such an analysis. Official ICT statistics report 97.1% of households with Internet access, 88.7% Internet use during the previous three months, and 26.5 million mobile subscribers. DataReportal estimates 18.19 million Internet users and 14.10 million social-media user identities in early 2024 (Bureau of National Statistics, 2025a,b; DataReportal, 2024). The institutional environment is also changing. Gambling law and the 2024 amendments, the law on online platforms and online advertising, and the governmental 2024–2026 plan address illegal gambling, betting advertising, and gambling-related harm; enforcement data record more than 10,000 websites advertising illegal gambling and betting entered for blocking and more than 1.6 thousand outdoor advertisements dismantled (Government of the Republic of Kazakhstan, 2024, 2025; Republic of Kazakhstan, 2007, 2023, 2024; Toleubekova and Osmanova, 2025). The Law on Microfinance Organizations and National Bank tables place the microfinance loan portfolio above KZT 1.5 trillion in 2025 (National Bank of Kazakhstan, 2025; Republic of Kazakhstan, 2012). Together, these indicators define the connected digital, credit, and regulatory environment in which gambling, online recruitment, and debt-producing financial access operate.

The analysis treats platform affordances, social legitimation networks, payment and credit infrastructures, and cognitive-affective reinforcement as a digital risk configuration that may facilitate entry and increase the perceived costs of exit. Digital mechanisms comprise platform-mediated visibility, access, payments, and re-entry. Social mechanisms concern legitimation through peers, family members, mentors, influencers, referrals, and closed chats. Cognitive mechanisms include reinforcement, perceived control, selective memory of wins, loss chasing, and risk perception. Shared cross-domain infrastructure and participant-level cross-domain evidence form separate analytic categories. Cross-domain entry requires a temporally ordered first participation in a second domain within one first-person case. Continued second-domain participation additionally requires repeated participation, consolidation, or escalation after entry into that domain. Migration requires explicit evidence of durable substitution, reorientation, or functional continuation across domains. Shared channels lacking a unique participant sequence constitute shared-infrastructure evidence.

Existing scholarship identifies several components of this regime. Research on online gambling explains the significance of constant availability, Internet-mediated access, gambling marketing, social-media exposure, operator advertising strategies, and digital payment affordances for the visibility, accessibility, and repeatability of gambling offers (Gainsbury, 2015; Guillou-Landréat et al., 2021; James and Bradley, 2021; Singer et al., 2024; Lakew et al., 2024). Moravec et al. (2025) further link social-media engagement to gambling severity. This literature establishes exposure and access mechanisms that may extend across adjacent digitally mediated products.

Scholarship on gamblified finance and pyramid-like schemes extends this mechanism account across adjacent products. Work on cryptocurrencies, crypto casinos, and gamified trading interfaces shows how financial language, immediacy, reward cues, transaction frequency, and perceived skill can frame risky participation as disciplined and controllable (Andrade and Newall, 2023; Packin et al., 2025; Leong Wei Lyn et al., 2025). Studies of pyramidal, Ponzi-like, and MLM-like schemes identify recruitment and retention structures based on kinship and status relations, displayed success, scarcity rhetoric, mentors, referral hierarchies, closed groups, and digital payments (Musaraj, 2011; Ryzhkova and Kashapova, 2023, 2024). These literatures identify shared channels, payment routines, social validation, and recovery-oriented narratives.

Kazakhstani scholarship provides the local context for this analysis. Existing studies examine gambling advertising and gambling-addiction propensity, the relationship between gambling revenues and harm reduction, gambling legislation, illegal gambling as a criminogenic factor, and the national gambling market (Buribayev et al., 2025; Buribayev and Khamzina, 2025; Toleubekova and Osmanova, 2025; Dzjazikova and Akhmedzhanova, 2024; Mukanov et al., 2025). These studies establish the relevance of gambling law, advertising, criminality, and market scale in Kazakhstan. The present analysis examines how channels, payment routes, referral structures, affective triggers, and recovery narratives operate across betting, online casinos, and pyramidal pseudo-investment schemes.

Across this literature, digital visibility, payment routines, social validation, and recovery narratives provide the analytical categories for examining betting, online casinos, and pyramidal pseudo-investment schemes.

The analysis asks when platform visibility and payment access gain social legitimacy, how reward, status, recovery expectations, and debt sustain participation, and how these mechanisms recur across gambling and pseudo-investment domains.

The present study connects these literatures through three sensitizing concepts. Rhodes’s risk environment framework locates harm in interactions among social, economic, institutional, politico-legal, and individual conditions (Rhodes, 2002, 2009). Platform affordances specify how digital services make particular actions more visible, easier, or more repeatable (Gibson, 1979; Norman, 2013; Bucher and Helmond, 2017). A practice-oriented perspective treats gambling and pseudo-investment participation as routinized configurations of devices, payments, social rituals, meanings, and affective dispositions (Bourdieu, 1977; Reckwitz, 2002; Shove et al., 2012). These concepts support a process framework of recurrent within-domain stages, recursive movement, optional post-crisis cross-domain entry, and contextual conditions within the digital risk configuration.

The three research questions follow from this mechanism-oriented problem. RQ1 examines how digital access, social validation, and cognitive-affective reinforcement shape entry and retention. RQ2 examines participant-level evidence for temporally ordered participation across domains and cross-source evidence of shared infrastructure. RQ3 addresses contextual conditions concerning recruitment, visibility, household recognition, and crisis response. Region and gender serve as descriptive source metadata where safely reportable.

RQ1. How do digital, social, and cognitive mechanisms shape entry into and retention within gambling and pseudo-investment schemes in Kazakhstan?

RQ2. Which participant-level records document temporally ordered participation across gambling and pseudo-investment domains, and which cross-source records document shared cross-domain infrastructure?

RQ3. What contextual boundary conditions concerning recruitment, visibility, household recognition, and crisis response can be illustrated from the available material?

The article makes two linked contributions. Empirically, it provides a case-level account of complete, partial, nonlinear, recursive, trial-only, same-domain re-entry, and cross-domain sequences in gambling and pseudo-investment participation. Theoretically, it integrates recurrent mechanisms across the corpus, graded participant-level temporal evidence, and shared cross-domain infrastructure documented through cross-source synthesis. The Results present case configurations, exposure-to-entry and retention mechanisms, sequential cross-domain evidence, contextual illustrations, and the associated metadata scope.

## 2 Materials and methods

### 2.1 Design and setting

A multi-source qualitative study in Kazakhstan combined in-depth interviews, expert interviews, and five online macro-regional focus groups. It examined entry, retention, crisis/exit, recursion, cross-domain participation, and contextual conditions in gambling-related and pyramidal pseudo-investment practices. Within-group coding, first-person case mapping, and provenance-preserving cross-source synthesis maintained source-specific analytic roles and mapped mechanisms within the purposive corpus.

The study followed a critical realist orientation. Respondents’ accounts were treated as situated narratives from which plausible digital, social, and cognitive mechanisms could be reconstructed, with attention to local context and to discordant, nonconsolidating, and recursive trajectories. The case-level analysis used five recurrent within-domain stages: exposure, entry, consolidation, escalation, and crisis/exit. Cross-domain entry was coded as an optional post-crisis event. Continued second-domain participation and durable migration were assessed through separate criteria.

The three research questions provided the primary analytic frame. The stages served as sensitizing categories within a heterogeneous process framework. Individual narratives retained their reported order, including skipped stages, direct movement to crisis/exit, same-domain re-entry, one-off cross-domain entry, and continued second-domain participation.

### 2.2 Participants by group and recruitment

#### 2.2.1 Recruitment strategy

Participants were recruited through anonymous announcements in social media and messengers, including relevant Telegram channels; professional contacts with practicing psychologists and civil society organizations; snowball referrals from interviewed participants; and formal institutional channels for law-enforcement officials. All participants were adults capable of providing informed consent. Exclusion criteria were absence or withdrawal of informed consent, acute mental-health decompensation at the time of contact, a foreseeable risk of deanonymization, and duplicate participation within the same data set. The archive records recruitment channels at group level and contains no route-specific counts of persons approached, screened, enrolled, or declining participation.

#### 2.2.2 Persons reporting gambling-related problems

This group comprised adults who reported participation in online betting, online casinos, or closely related gambling products during the year preceding the interview and described debt, household conflict, concealment, loss of control, psychological distress, help-seeking, or other gambling-related harms. Eligibility rested on self-reported participation and harm. The protocol used no DSM-5 diagnostic assessment, Problem Gambling Severity Index (PGSI), or South Oaks Gambling Screen (SOGS). The manuscript designates this group as “persons reporting gambling-related problems” or “persons reporting gambling-related harms.”

#### 2.2.3 Participants in financial pyramid schemes

This group comprised adults who had made an actual financial contribution to a pyramidal pseudo-investment, Ponzi-like, or MLM-like scheme, including schemes presented as investment packages, trading clubs, personal account systems, or referral-based projects. Eligibility required reported participation in a referral, mentor, or closed-chat recruitment structure. Participants remained eligible after a financial loss or a temporary payout. Cases were retained when participants could specify at least an approximate period of involvement. The interview guide recorded the period of participation, mode of entry, payment route, presence or absence of referral recruitment, experience of withdrawals or losses, and exit or crisis circumstances.

#### 2.2.4 Family members or co-residents affected by gambling-related practices

This group comprised adults who shared a household, family budget, or sustained care relationship with a person involved in gambling and had directly observed financial, relational, psychological, or legal consequences. The public data contain no reliable participant-family linkage. Family and co-resident interviews were analyzed as a separate collateral source group. Their accounts informed interpretation of harm, concealment, crisis recognition, and household intervention; participant chronology and transition counts were based on first-person cases.

#### 2.2.5 Practicing psychologists and psychotherapists

Professional participants had direct counselling, clinical, rehabilitation, crisis-support, university-service, or private-practice experience with gambling-related harms, loss chasing, debt-related distress, family conflict, or financial-pyramid-related psychological distress. The term “expert” denotes direct professional case experience. Interviews recorded professional setting, approximate years of practice, and types of cases where available.

#### 2.2.6 Law-enforcement officials

Institutional experts held functional roles related to financial fraud, illegal gambling, cybercrime, economic investigations, or regional enforcement units. Public reporting retains functional roles and institutional perspectives and omits personal ranks, offices, and identifying operational details.

#### 2.2.7 Focus groups

Five online focus groups were conducted across the southern, western, northern, central, and eastern macro-regions, with 6–8 participants per session. These mixed adult community groups discussed personal, family, or close-community exposure to gambling and pseudo-investment practices, including advertising encounters, household consequences, local normalization, and coping or resistance strategies. The focus-group session served as the analytic and reporting unit, with one session per macro-region.

The archive contained reportable regional metadata for 20 of the 44 first-person cases. For the remaining 24, regional information was unavailable, insufficiently specific, or suppressed during de-identification. It contained no complete group-level distributions for urban–rural residence, age, gender, recruitment route, interview language, collection mode, or interviewer assignment. Table 1 lists the documented metadata. Region and gender served solely as descriptive source metadata.

**Table 1:** Profile of qualitative data sets, recruitment, collection modes, documented metadata, and analytic roles.

Source group and N	Recruitment and collection	Available characteristics and metadata	Analytic role and reporting scope
Persons reporting gambling-related problems (*n* = 25)	Mixed announcements, professional/civil-society contacts, and snowball referrals; online or in person; Russian/Kazakh; usually 50–90 min.	Adults; selected urban residence records; region reportable for 12/25. Group-level age, gender, mode, language, route, interviewer, and complete urban–rural distributions unavailable.	D evidence; included in the 44-case trajectory denominator.
Pseudo-investment participants (*n* = 19)	Mixed announcement, professional/civil-society, and referral channels; online or in person; Russian/Kazakh; usually 50–90 min.	Adults; actual contribution and approximate participation period required; region reportable for 8/19. Other group-level distributions unavailable.	D evidence; included in the 44-case trajectory denominator.
Family members or co-residents (*n* = 22)	Mixed community, professional/civil-society, and referral channels; online or in person; Russian/Kazakh; usually 50–90 min.	Adults with a household, budget, or sustained-care relationship; region reportable for 10/22; public reporting at collateral-group level.	Separate C evidence; excluded from first-person trajectories.
Psychologists or psychotherapists (*n* = 22)	Professional contacts; online or in person; Russian/Kazakh; usually 50–90 min.	Direct relevant practice experience; setting, approximate experience, and case types recorded where available; region reportable for 11/22.	X evidence; professional mechanism interpretation.
Law-enforcement officials (*n* = 12)	Formal institutional channels; online or in person; Russian/Kazakh; usually 50–90 min.	Functional relevance to fraud, illegal gambling, cybercrime, economic investigation, or enforcement; region reportable for 4/12.	X evidence; institutional and infrastructure context.
Macro-regional focus groups (5 sessions; 6–8 participants each)	Mixed adult community recruitment; online; Russian/Kazakh; approximately 80–90 min; one session per macro-region.	Session-level composition only; no individual-participant N or complete age/gender distribution.	X evidence; session-level contextual coverage.

Recruitment routes may overlap. NA denotes a macro-regional identifier that was undisclosed, insufficiently specific, or suppressed during de-identification. The direct first-person trajectory denominator comprises 44 substantive interviews: 25 gambling-related and 19 pseudo-investment cases. One unused blank row in the gambling source workbook was excluded. Family, professional, institutional, and focus-group materials retained separate evidentiary roles. The five focus groups provide contextual coverage through one session per macro-region.

### 2.3 Data collection

Data collection took place in Russian and/or Kazakh during May–June 2025, after institutional ethics approval (Protocol No. 2709, 02 December 2024). Participants chose either language and could code-switch to reflect ordinary speech. Transcription and de-identification retained these language choices.

Before each interview or focus group, participants completed signed written informed consent or audio-recorded verbal informed consent under the approved protocol. Interviews and focus groups were transcribed, de-identified, and stored under restricted access. Any compensation was fixed and independent of response content.

Z. K. supervised recruitment and fieldwork. All interviewers followed the approved semi-structured guides, informed-consent procedures, distress-sensitive interviewing protocol, and confidentiality requirements. The de-identified audit contains author-level fieldwork records and no interview-by-interviewer assignments. The analysis excluded interviewer strata.

Individual interviews were semi-structured, usually lasted 50–90 min, and were conducted online or in person according to participant availability and safety considerations. Russian and Kazakh were used according to participant preference. The de-identified archive contains no source-group distributions of mode, language, or interviewer assignment. Interviews with persons reporting gambling-related harms addressed exposure, entry, routines, debt, concealment, attempts to stop, other risk products, and crisis or exit through a general other-risk-products prompt. Interviews with pseudo-investment participants used a 17-question guide covering trust, mentors and referrals, emotional triggers, initial payouts, losses, reinvestment, withdrawal delays, and exit. Its final substantive question systematically elicited later betting, casino, cryptocurrency, or trading activity. Direction-specific elicitation was systematic for P → G and general for G → P.

Interviews with practicing psychologists and psychotherapists focused on clinical and counselling observations related to illusion of control, loss chasing, delayed help-seeking, family involvement, debt-related distress, and relapse risk. Interviews with law-enforcement officials documented functional observations on illegal gambling, fraud infrastructures, shared online channels, mirror sites, closed chats, payment routes, product repackaging, and barriers to enforcement.

The five focus groups were conducted online and lasted approximately 80–90 min each. A common guide covered personal and family exposure, advertising and social-media channels, community normalization, perceived consequences, help-seeking barriers, and coping or resistance strategies.

### 2.4 Data analysis

The primary analytic approach was abductive, codebook-oriented thematic analysis within the critical realist orientation described in Section 2.1. It supported systematic identification of patterned meanings across a heterogeneous qualitative corpus through theoretically informed and data-driven coding (Braun and Clarke, 2006; Fereday and Muir-Cochrane, 2006; Nowell et al., 2017). Critical realism guided the reconstruction of plausible generative mechanisms from situated accounts within the purposive corpus (Sayer, 1992; Danermark et al., 2002; Fletcher, 2017).

The analytic procedure combined codebook-oriented thematic analysis with iterative coding, memo-writing, and negative-case analysis from constructivist grounded theory (Charmaz, 2014) and temporal ordering of within-case narratives from narrative analysis (Riessman, 2008). First- and second-cycle coding terminology followed Saldaña (2021).

First-cycle coding identified fragments concerning exposure channels, entry triggers, reinforcement, debt, referrals, concealment, crisis, exit, re-entry, and possible cross-domain entry. Second-cycle pattern coding linked these fragments to digital, social, and cognitive mechanism classes and to five recurrent within-domain stages. The framework developed abductively. Exposure, entry, retention, cross-domain participation, and exit served as initial sensitizing domains. Temporal ordering produced distinct exposure, entry, consolidation, and escalation codes. Negative-case analysis located cross-domain entry as optional and crisis/exit as potentially temporary.

Before cross-source thematic synthesis, a case-by-stage matrix was constructed for all 44 first-person accounts: 25 gambling-related and 19 pseudo-investment cases. Each row represented one focal participant. Stage status was recorded as present, not documented/not ascertainable, or explicitly denied where the source permitted that distinction. An adjacent transition required two consecutive stages in the published within-case sequence. Repeated episodes were retained as recursion, and each participant contributed at most one count to a given transition.

Evidence provenance was coded separately. D denoted direct temporal ordering within a first-person case. C denoted an unlinked family or co-resident collateral account concerning a gambling-related focal person. X denoted cross-case or cross-source mechanism evidence. Participant chronology and counts derived exclusively from D evidence. C and X evidence informed corroboration, qualification, challenge, and mechanism scope. Cross-domain classification proceeded through XDE_DIRECT for temporally ordered second-domain entry, XDE_CONT for repeated participation, consolidation, or escalation in the second domain, and MIG_STRONG for explicit durable substitution, reorientation, or functional continuation. Shared channels, administrators, payment routes, recruitment routines, mirror solutions, and recovery narratives lacking a unique participant sequence were coded as shared-infrastructure evidence.

#### 2.4.1 Cross-source integration and handling of discordant interpretations

Analytic integration was sequential. Each respondent group was coded within source type using the shared codebook. The 44 first-person accounts were mapped case by case to establish stage presence, narrated order, adjacent transitions, recursion, bypasses, and graded cross-domain classifications. Family, psychologist or psychotherapist, law-enforcement, and focus-group material was then integrated with the direct-case matrix through a joint display organized by research question and model element. Each source relation was classified as convergence, complementarity, qualification, discordance, or absence of relevant evidence. This sequence preserved the distinct empirical objects addressed by each source group.

The source groups had distinct inferential roles. D evidence established participant chronology and case counts. C evidence addressed concealment, debt, crisis recognition, and household intervention. X evidence identified cross-case mechanisms, institutional infrastructures, and contextual conditions. Each model element was reported at its highest supported provenance level. Overlapping channels described by law enforcement and focus groups were classified as shared cross-domain infrastructure. Participant migration required D-level evidence meeting the durable criterion.

Cross-source discordance and intercoder disagreement were handled through separate procedures. When first-person chronology differed from a family, professional, institutional, or community interpretation, both source positions were retained. D evidence governed participant temporal sequence. The other source qualified visibility, harm recognition, or mechanism scope. Items with unresolved source-language order or stage presence remained not documented/not ascertainable. Intercoder differences were resolved through source-language reinspection, application of the operational definition and provenance rule, and consensus. Supplementary material S5 and the workbook audit sheets document these integration and adjudication rules.

The research questions structured second-cycle analysis. Exposure, entry, consolidation, escalation, reinforcement, perceived control, debt, referrals, and concealment addressed RQ1. Direct cross-domain entry, continued second-domain participation, durable migration, and cross-source evidence of shared infrastructure addressed RQ2. Peer mediation, platform visibility, household recognition, family or religious intervention, and limiting cases addressed RQ3 and the boundary conditions of RQ1 and RQ2. Region and gender served as descriptive source metadata.

Negative cases were retained in their narrated order. Trial-only participation, single-domain trajectories, bypassed stages, offline-first escalation, temporary exit, same-domain re-entry, one-off cross-domain trial, and continued post-crisis second-domain participation were treated as empirical configurations. Newly assigned de-identified case IDs G01-G25 and P01-P19 were used for the case-level audit in Supplementary material S3, Supplementary data S1.

Mutually exclusive configurations were assigned hierarchically. Cases with an alternative order, same-domain re-entry, or cross-domain entry were classified as nonlinear/recursive/alternative. Remaining cases with entry followed by crisis/exit and no documented consolidation were classified as trial-only/nonconsolidating. Cases with the exact EXP → ENT → CON→ESC → CRS order and no recursion were classified as ordered single-domain five-stage. All remaining orderable cases were classified as other partial ordered. The tested six-element specification EXP → ENT → CON→ESC → XDE → CRS occurred in no case.

Table 2 presents a compact analytic codebook with explicit research-question alignment. Supplementary material S1 provides the full codebook with operational definitions, indicators, example identifiers, stages, mechanism classes, and research-question mapping.

**Table 2:** Compact analytic codebook with explicit research-question alignment (full codebook in Supplementary Material S1).

Stage/analytic domain	Mechanism	Example code	Research question	Analytic role
Exposure	Digital/social visibility and legitimation	EXP_ALG; EXP_SOC	RQ1	Detects platform visibility coupled with proximate social proof.
Entry	Low-barrier trial and perceived control	ENT_BONUS	RQ1	Marks trial entry through bonuses, free bets, symbolic bets, trial accounts, or small contributions.
Consolidation	Cognitive routinization and status attachment	CON_COG; RET_STATUS	RQ1	Traces routine formation through perceived control, ranks, statuses, referrals, and repeated participation.
Escalation	Loss recovery and debt amplification	ESC_CHASE; ESC_DEBT	RQ1	Captures chasing, borrowing, microloans, reinvestment, package upgrades, and materially intensified participation.
Participant-level cross-domain evidence	Sequential entry, continuation, and strong migration criterion	XDE_DIRECT; XDE_CONT; MIG_STRONG	RQ2	Separates any temporally ordered second-domain entry from continued participation and from explicit durable substitution or reorientation.
Shared cross-domain infrastructure	Shared channels, access, payments, and recovery narratives	PORT_XDOMAIN; PORT_ACCESS	RQ2	Reports shared infrastructure as X evidence when no unique participant transition can be reconstructed.
Contextual source metadata	Selected contextual illustrations; region and gender as descriptive metadata only	CTX_REGION; CTX_GENDER	RQ3	Links specific contextual mechanisms to identified sources; region and gender remain descriptive metadata.
Crisis/exit, re-entry, and limiting cases	External interruption, access restriction, recursion, and nonconsolidation	CRS_FAMILY; CRS_SELFEX; REENTRY; LIM_SINGLE	RQ3; RQ1-RQ2 boundaries	Captures family intervention, self-exclusion, help-seeking, trial-only entry, same-domain re-entry, and temporary or non-durable exit.

Table 2 lists analytic codebook labels. Table 4 defines the separate quotation identifiers used in the Results, including the format [TYPE-REGION-GENDER, AGE].

Regional and gender metadata were reviewed after case-level trajectory coding. Region was reportable for 20 of 44 first-person cases and unavailable or suppressed for 24. The focus-group component provided one session in each macro-region. Selected accounts illustrate source-specific contextual mechanisms. Region and gender remain descriptive metadata throughout the analysis.

### 2.5 Trustworthiness

Trustworthiness was addressed through credibility, dependability, confirmability, and transferability criteria for qualitative inquiry and thematic analysis (Lincoln and Guba, 1985; Nowell et al., 2017). The analysis used interlevel triangulation, analytic memoing, group-specific code-stability review, double coding across respondent groups, negative-case analysis, provenance-preserving joint displays, and an audit trail. Approximately 23% of the 100 individual interviews across five source groups were double coded. The public audit records the corpus-level proportion. It contains no group-specific allocation or formal agreement coefficient. Discrepancies were resolved through consensus review.

Y. B. led the codebook-oriented analytic structure, formal analysis, case mapping, and construction of the audit displays. Z. K. led fieldwork supervision and source-level validation. Both authors reviewed double-coded discrepancies, disputed interpretations, negative cases, and the final framework. They resolved differences by re-reading the source-language segment, applying the code definition, provenance class, and analytic memo, and reaching consensus. Ambiguous material was coded as not documented/not ascertainable. All 44 first-person case maps were checked against source-language records for denominator accuracy, stage presence, narrated order, adjacent links, bypasses, recursion, and evidence provenance.

Analytic sufficiency was assessed within each respondent group through recurrence and stability of first-cycle code definitions and continued review for negative, alternative, and boundary cases. Cross-source synthesis then assessed convergence, complementarity, qualification, discordance, and absence of evidence. Supplementary Table S5c reports group-specific code stability and analytic purpose. The archive contains no contemporaneous interview-by-interview stopping logs or numerical saturation thresholds. Later materials elaborated or recombined established first-cycle codes. Negative cases refined framework boundaries. The five focus groups provided contextual coverage across macro-regions.

The audit structure had two levels. The restricted internal audit retained source-record identifiers, source-language verification materials, linkage for source-level validation, respondent type, safely reportable metadata, stage presence, adjacent transitions, configuration type, cross-domain classification, provenance, and analytic memos. Public Supplementary data S1 is confined to public case IDs and de-identified analytic variables. The two cross-domain cases and the selected same-domain reference case were verified against restricted source-language records. Supplementary material S5, Supplementary data S1 provide public joint displays for source integration, discordance handling, code stability, research-team roles, and available recruitment and data-collection metadata.

Interviews and focus groups were conducted in Russian and/or Kazakh, and translation formed part of the analytic audit trail. Coding used de-identified source-language transcripts. English translation was prepared for excerpts selected for publication following recommendations for transparency in cross-language qualitative research (Temple and Young, 2004; Squires, 2009). A bilingual researcher prepared the forward translation, and a second bilingual researcher verified semantic accuracy, pragmatic force, and contextual equivalence against the original excerpt. Disagreements, idiomatic expressions, and culturally specific terms were resolved through consensus and recorded in translation memos where necessary. The English excerpts prioritize conceptual equivalence with participants’ meanings. Original-language excerpts, English translations, and translation notes were retained under the same anonymized identifiers used in the audit trail.

Quotations used in the Results illustrate key codes and mechanisms and preserve diversity of sources. All quoted excerpts are author translations from Russian or Kazakh source-language transcripts prepared according to the procedure described above. Quotations were anonymized by removing personal, institutional, and geographic identifiers. Published excerpts are followed either by anonymized quotation identifiers of the form [TYPE-REGION-GENDER, AGE] or by public case IDs when case-level analytic provenance is required. The key to quotation identifiers is provided at the beginning of the Results section; demographic elements are reported only where disclosure does not create a foreseeable risk of re-identification.

### 2.6 Ethics

The study involved adult participants. The Scientific Ethics Committee of the Non-Profit Joint-Stock Company “Zhetysu University named after Ilyas Zhansugurov” reviewed and approved the research protocol, recruitment and informed-consent procedures, distress-sensitive interviewing, anonymization, and data-protection arrangements before recruitment and data collection (Protocol No. 2709, 02 December 2024). Fieldwork followed in May–June 2025.

Under the approved dual-modality procedure, participants completed signed written informed consent or audio-recorded verbal informed consent before participation. The consent record documented voluntary participation and the right to decline questions, pause, or withdraw. Transcripts were de-identified before analysis; audio recordings, transcripts, source documents, and linkage files remained restricted to the research team. The manuscript reports de-identified quotations and aggregate or descriptive materials. It contains no identifiable personal information, so separate consent for publication of such information was inapplicable.

Because the study involved persons reporting gambling-related harms, debt-related distress, and participation in potentially stigmatized or illegalized practices, data collection followed distress-sensitive procedures. Interviewers used neutral prompts, avoided requests for identifying operational details that could increase legal, social, or psychological risk, and did not pursue participation where acute distress or immediate safety concerns were apparent. In such cases, respondents were encouraged to seek appropriate psychological, medical, legal, or crisis-support assistance.

## 3 Results

### 3.1 Case-level distribution and the cross-case process framework

A case-by-stage audit was completed for all 44 first-person accounts: 25 gambling-related cases and 19 pseudo-investment cases. All 44 contained at least one orderable adjacent link. No case documented the complete EXP → ENT → CON→ESC → XDE → CRS order. Eleven cases followed the exact five-stage within-domain order EXP → ENT → CON→ESC → CRS; 17 contained other partial ordered sequences; 12 showed nonlinear, recursive, or alternative movement; and 4 were trial-only or nonconsolidating. The categories were assigned by the hierarchy specified in Section 2.4, are mutually exclusive, and describe analytic coverage within the purposive corpus (Table 3).

**Table 3:** Case-level support for the cross-case process framework.

Case-level configuration	G	P	Total	Operational rule/interpretation
Panel A. Mutually exclusive first-person case configurations				
Complete tested six-element order	0	0	0	Tested EXP → ENT → CON→ESC → XDE → CRS specification within one first-person case.
Ordered single-domain five-stage	7	4	11	Exact EXP → ENT → CON→ESC → CRS with no recursion or cross-domain entry.
Other partial ordered	11	6	17	Remaining orderable cases after hierarchical assignment; exact five-stage chronology incomplete.
Nonlinear, recursive, or alternative	6	6	12	Alternative order (1), same-domain re-entry (9), or post-crisis cross-domain entry (2).
Trial-only or nonconsolidating	1	3	4	Entry followed by crisis/exit with no documented consolidation or re-entry.
Total first-person cases	25	19	44	One focal participant per row in the case-level matrix.
Panel B. Adjacent within-case transition coverage, recursion, and cross-domain classification				
Adjacent transition/audit item	G	P	Total	Evidence interpretation
EXP → ENT	16	19	35	Exposure and first participation were adjacent in the case sequence.
ENT → CON	23	16	39	Entry and consolidation were adjacent; P04 contributes through ENT(G) → CON(G).
CON→ESC	21	9	30	Consolidation and escalation were adjacent; P04 contributes through CON(G) → ESC(G).
ESC → CRS	19	8	27	Escalation and crisis/exit were adjacent in the case sequence.
CON → CRS	1	7	8	Adjacent link; seven cases contained no documented escalation. G06 contained escalation earlier in the episode.
ENT → ESC → CON alternative order	1	0	1	G06 escalated at entry and then consolidated before crisis/exit.
ENT → CRS without CON	1	4	5	Entry proceeded directly to crisis/exit without documented consolidation.
CON → CRS without documented ESC	0	7	7	Subset of CON→CRS with no escalation documented in the relevant episode.
Any bypass of an intermediate stage	2	11	13	Unique cases across the three bypass configurations above.
CRS → ENT/CON same-domain re-entry	5	4	9	Crisis/exit was followed by renewed participation in the same domain.
CRS → XDE → ENT(G), P → G	0	2	2	Two temporally ordered entries into gambling followed pseudo-investment crisis.
ENT(G) → CON(G) continued second-domain participation	0	1	1	P04 continued beyond a trial into repeated gambling.
CON(G) → ESC(G) continued second-domain participation	0	1	1	P04 subsequently escalated in gambling.
Cross-domain sequence ending after ENT(G)	0	1	1	P17 reported one lottery purchase without documented continuation.
Confirmed durable migration	0	0	0	No case documented explicit durable substitution, reorientation, or functional continuation across domains.
ESC → XDE	0	0	0	No first-person case supported cross-domain entry before crisis/exit.
XDE → CRS	0	0	0	No first-person case supported cross-domain entry followed by crisis/exit in the second domain.

G = first-person gambling-related cases; P = first-person pseudo-investment cases. Panel A categories are mutually exclusive and sum to 44. Panel B reports unique cases for adjacent links and specified bypass or cross-domain audits; rows are not additive. ENT(G) → CON(G) and CON(G) → ESC(G) are normalized into the overall ENT→CON and CON→ESC counts. Participant-level sequences and counts derive exclusively from D-level first-person evidence.

Across cases, adjacent EXP → ENT was documented in 35 accounts, ENT → CON in 39, CON→ESC in 30, and ESC → CRS in 27. Eight cases contained an adjacent CON→CRS link. Seven pseudo-investment cases contained no documented escalation in the relevant episode. G06 contained escalation before consolidation in the alternative order ENT → ESC → CON→CRS. Thirteen unique cases bypassed at least one intermediate stage, and nine showed same-domain re-entry after crisis/exit. Two cases contained CRS → XDE → ENT(G). P04 continued through ENT(G) → CON(G) → ESC(G). P17 ended after one documented gambling entry. The strong migration criterion was met in zero cases; ESC → XDE and XDE → CRS also occurred in zero cases.

Evidence provenance was recorded separately. First-person accounts supplied chronology and case counts. Family accounts informed later-stage harm and intervention. Psychologist, law-enforcement, and focus-group materials informed mechanisms and shared infrastructure at cross-source level.

The provenance-preserving joint display assigned source-specific support. C and X material informed interpretation of platform visibility, social legitimation, routinization, debt, crisis recognition, and shared infrastructure. D evidence established two P → G entries and zero cases of durable substitution. Each claim in the cross-case synthesis retains its source-specific provenance.

**Figure 1:**
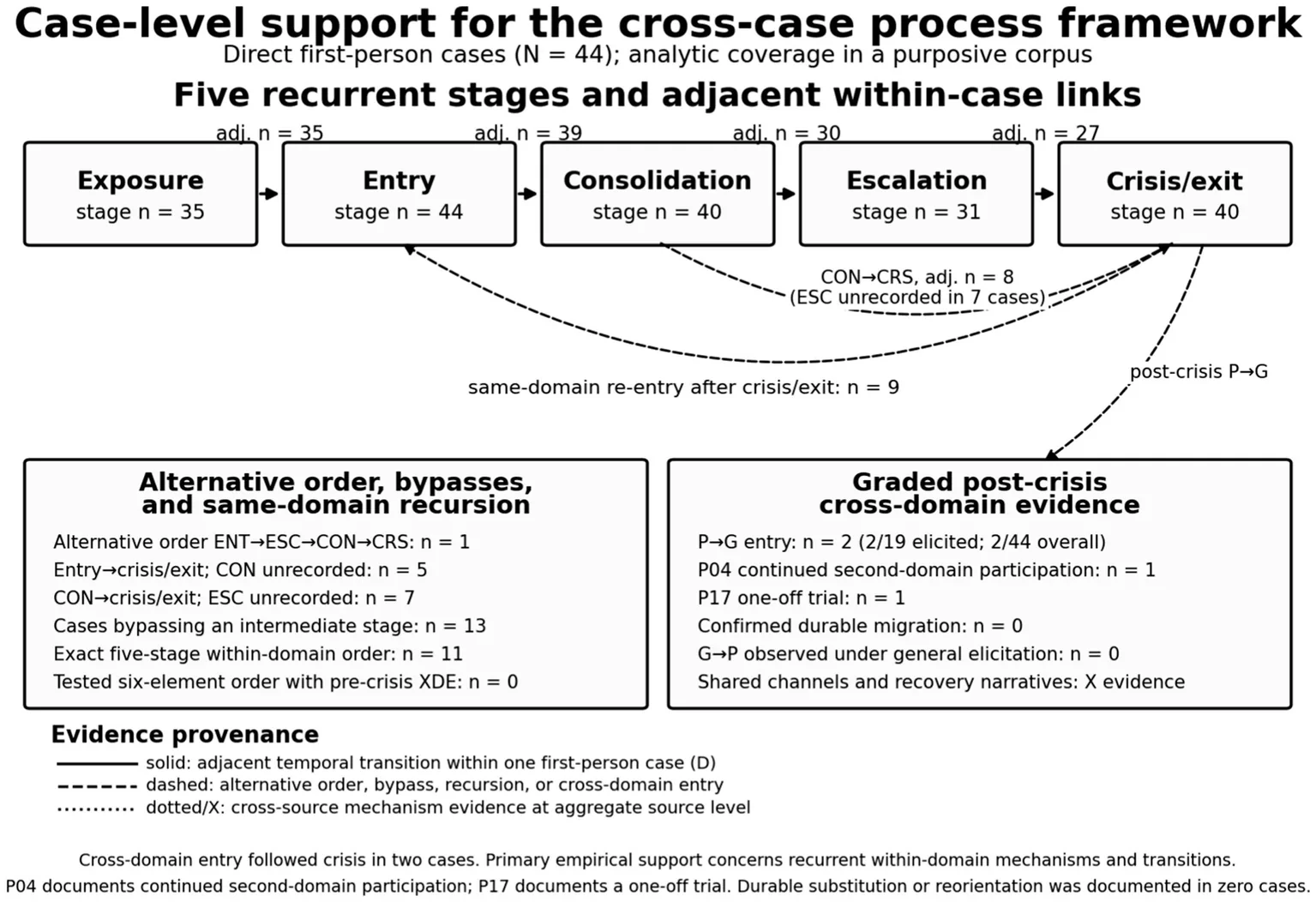
Case-level support for the cross-case process framework. D denotes an adjacent temporal transition documented within one first-person case. Solid arrows show the principal within-domain links; dashed arrows show alternative, bypass, recursive, or post-crisis cross-domain movement. X denotes cross-source mechanism evidence at aggregate source level. Counts are unique case counts and represent analytic coverage in the purposive first-person corpus. Among the 19 pseudo-investment interviews with systematic adjacent-risk elicitation, two P → G entries were documented; these were also two of the 44 first-person cases overall. One case showed continued second-domain participation and one remained a one-off trial; durable migration occurred in zero cases.

Figure 1 presents the cross-case process framework. The upper line reports adjacent within-domain links. The lower paths display alternative order, bypasses, same-domain recursion, and graded post-crisis cross-domain evidence. Sections 3.2 and 3.3 address within-domain links. Section 3.4 defines one-off entry, continued second-domain participation, durable migration, and shared cross-domain infrastructure as separate categories. Sections 3.5 and 3.6 present contextual illustrations and limiting cases.

All excerpts quoted in the Results are English author translations from Russian or Kazakh transcripts; Section 2.5 describes translation and verification. Illustrative quotations retain the anonymized format [TYPE-REGION-GENDER, AGE]. Case-level statements and cross-domain excerpts use audit IDs G01-G25 and P01-P19. These public analytic labels are independent of restricted source-workbook row labels. Region and gender elements serve as descriptive metadata. Table 4 provides the complete key.

**Table 4:** Key to quotation and case identifiers used in the Results.

Element in identifier	Values used in the manuscript	Meaning
TYPE	IG	Individual interview with a person reporting gambling-related problems or harms.
TYPE	FP	Individual interview with a participant in a pyramidal pseudo-investment, Ponzi-like, or MLM-like scheme.
TYPE	FM	Individual interview with a family member or co-resident affected by gambling-related practices.
TYPE	PSY	Interview with a practicing psychologist or psychotherapist.
TYPE	LE	Interview with a law-enforcement official or institutional expert.
TYPE	FG	Focus-group excerpt. Where safely reportable, the identifier refers to the quoted speaker’s metadata. The focus-group component is reported as macro-regional session material, with the session as the analytic unit.
REGION	S	Southern macro-region.
REGION	W	Western macro-region.
REGION	N	Northern macro-region.
REGION	C	Central macro-region.
REGION	E	Eastern macro-region.
REGION	C/E or other compound regional values	Aggregated macro-regional category used where exact regional disclosure could increase re-identification risk. Compound values denote deliberate aggregation, not missing data.
REGION	NA	Region not disclosed, not recorded with sufficient specificity, or suppressed during de-identification to prevent deanonymization.
GENDER	M	Male.
GENDER	F	Female.
GENDER	NA	Gender not safely reportable or not recorded.
AGE	Number	Age in years at interview or focus-group participation, where safely reportable.
AGE	Age range	Age band used where exact age could increase re-identification risk.
AGE	NA	Age not safely reportable or not recorded.
Examples	[FG-S-M, 19]	Male focus-group speaker aged 19 from the southern macro-region.
Examples	[IG-C/E-M, 24]	Male interviewee aged 24 reporting gambling-related harms from an aggregated central/eastern macro-regional category.
Examples	[LE-NA-M, 38]	Male law-enforcement official or institutional expert aged 38 whose region was not disclosed, not recorded with sufficient specificity, or suppressed for de-identification.
Examples	[PSY-NA-F, 43]	Female psychologist or psychotherapist aged 43 whose region was not safely reportable or not recorded.
Examples	[FP-C/E-F, 30]	Female pseudo-investment-scheme participant aged 30 from an aggregated central/eastern macro-regional category.
Examples	[FG-C/E-F, 25–34]	Female focus-group speaker in the 25–34 age band from an aggregated central/eastern macro-regional category.
CASE ID	G01-G25	Newly assigned de-identified IDs for the 25 substantive first-person gambling-related cases used in Table 3, Supplementary Material S3.
CASE ID	P01-P19	Newly assigned de-identified IDs for the 19 first-person pseudo-investment cases used in Table 3, Supplementary Material S3.

### 3.2 RQ1, entry component: from exposure to entry—digital access and social validation

The exposure-to-entry transition was directly documented in 35 of the 44 first-person cases. Respondents commonly described initial participation in which platform-mediated visibility, instant access, bonuses, free bets, or small first deposits gained credibility through proximate or trusted social sources. Trial participation was narrated as a normalized, low-commitment experiment.

“In TikTok it appears as ordinary clips: predictions, ‘easy money,’ stories of ‘how I made a profit.’ You do not search for anything; it simply shows up in the feed. Between memes and music videos there is an ‘install’ button, and it feels as if this is a normal thing.” [FG-S-M, 19]

“I downloaded the app from a blogger’s link; they promised a free bet for registration. I placed a bet ‘on my favorite team,’ and it immediately showed a gain. You get the feeling that you understand how it works and you want to continue.” [IG-C/E-M, 24]

“The first contact now is almost always on the phone. Young people aged 16 to 25 come in through social networks and messengers; the family sees only the consequences. The online format makes participation latent. The points of entry are personal devices and closed chats.” [LE-NA-M, 38]

At the coding level, EXP_ALG, EXP_SOC, and ENT_BONUS formed a recurrent exposure-and-validation configuration: platform-amplified visibility, social proof from friends, family members, bloggers, mentors, or closed chats, and a low-commitment trial mechanism. EXP → ENT was documented in all 19 pseudo-investment cases and 16 gambling-related cases. Other gambling-related accounts began after entry or contained insufficient temporal detail for an exposure-to-entry link. Distrust toward the recruiting channel or visible stories of bankruptcy, debt, or deception sometimes confined participation to a single episode. In these accounts, initial participation combined platform-mediated exposure with social validation.

### 3.3 RQ1, retention component: from consolidation to escalation—cognitive and debt-related retention mechanisms

The case-level audit documented adjacent ENT → CON in 39 cases and CON → ESC in 30. Gambling accounts linked continued participation to occasional wins and loss chasing. Pseudo-investment accounts linked it to credited accruals, reinvestment, package levels, rank, and referral status. Across the two domains, reward and recovery narratives supplied reasons for repeated payments or participation.

“After a series of losses, I start chasing them. I convince myself that one more try will recover the money. I increase the stake, switch markets, and take out a microloan ‘to cover the debt.’ A couple of successful bets in a row feel like proof of the ‘right strategy.’ The overall loss continues to grow.” [IG-W-M, 33]

“In the ‘personal account’ they explained: ‘Increase the package and the next percentage level will open.’ I reinvested every small payout and thought I was ‘recovering the loss.’ When delays started, I added another <amount redacted> ‘to break even’ and preserve my rank. The debt increased.” [FP-C/E-F, 30]

“Loss chasing is a common mechanism. After losses, the impulse to recover money increases. Variable reinforcement provides occasional wins; accessible microloans and encouragement in chats lead to further participation. In pyramids, reinvestment and package upgrades sustain participation and contribute to escalation.” [PSY-NA-F, 43]

These excerpts align with the adjacent transition from consolidation to escalation, documented in 30 cases. Participants’ accounts centered on recovery, rank preservation, or avoidance of acknowledged loss. Harmful participation produced debt. Participants cited existing debt as a reason for additional betting, reinvestment, recruitment, or package upgrading. The excerpts document narrative co-occurrence. Estimating independent causal effects requires prospective evidence. Adjacent ESC → CRS was documented in 27 cases. Eight cases contained adjacent CON→CRS, including seven pseudo-investment cases with no documented escalation and G06, in which escalation preceded consolidation.

### 3.4 RQ2: graded participant-level evidence of post-crisis cross-domain entry

Among the 19 pseudo-investment participants with systematic elicitation of later betting, casino, cryptocurrency, or trading activity, two cases documented gambling entry after pseudo-investment crisis. These cases also represented two of the 44 first-person cases in the full trajectory corpus. P04 progressed to repeated gambling and escalation. P17 reported one lottery purchase. The corpus contains two sequential P → G entries, one case of continued second-domain participation, and zero cases meeting the durable-migration criterion. These counts indicate evidentiary coverage within the purposive qualitative corpus. XDE_DIRECT required second-domain entry after participation in the first domain; XDE_CONT required repeated participation, consolidation, or escalation in the second domain; MIG_STRONG required explicit durable substitution, reorientation, or functional continuation across domains.

“I tried lotteries and betting. There were some winnings; once I won a very large amount. I wanted to continue after that.” [P04]

P04 entered the pseudo-investment scheme through a trusted coworker and expected passive returns. After one contribution produced a loss, the organizers proposed a second account and another contribution. She considered the proposal briefly and refused. She later tried lotteries and betting. The published excerpt captures entry into the second-domain activity. The verified source-language record additionally documented repeated gambling and further losses, supporting CRS → XDE → ENT(G) → CON(G) → ESC(G). This case meets the continued-participation criterion. The durable-migration criterion remains unmet because the account contains no explicit durable substitution, reorientation, or functional continuation across domains.

“After all of this, I still thought, ‘What if I get lucky?’ I bought one lottery ticket.” [P17]

P17 entered through a multi-step social referral and promotional brochures. An early payout prompted further investment; the subsequent collapse produced a major loss. After that crisis, the participant bought one lottery ticket while expressing a recovery expectation. The record contains no repeated gambling, consolidation, or escalation. The sequence CRS → XDE → ENT(G) is classified as one-off sequential participation.

P19 provides a post-crisis same-domain case. The participant entered new pyramid schemes after earlier collapses, following trusted trainers and acquaintances and participating in referral, status, and larger-package structures. Same-domain re-entry occurred in nine cases. Cross-domain entry occurred in two cases, including one case of continued second-domain participation.

Direct records contained zero G → P cases. The pseudo-investment guide systematically elicited later betting, casino, cryptocurrency, and trading activity. The gambling guide used a general other-risk-products prompt. P → G reporting uses the 19-case direction-specific elicitation set and the 44-case overall corpus. The G → P count represents directly observed cases under general elicitation.

Cross-source materials documented shared infrastructure across domains. Law-enforcement interviews described the same Telegram channels, bots, advertising techniques, and rapid payment arrangements being used for betting and pseudo-investment or cryptocurrency schemes; one institutional account described a channel that first promoted sports betting and later promoted cryptocurrency investments. Focus-group material identified Instagram, TikTok, and Telegram as overlapping recruitment environments. These observations are classified as X evidence and document shared use of channels and recovery narratives. They do not increase the participant-level cross-domain count.

### 3.5 Contextual illustrations and metadata scope

Regional information was reportable for 20 of the 44 first-person cases and unavailable or suppressed for 24. The four illustrations below address intergenerational normalization, peer escalation, household visibility, and recruitment. Regional identifiers and gender markers serve as de-identified source metadata; the analytic unit is the contextual mechanism illustrated by each account (see Table 5).

**Table 5:** Selected contextual illustrations and explicit inferential restrictions.

Contextual mechanism	Illustrative account and source	Illustrative evidence	Analytic scope
Intergenerational normalization and crisis recognition	One focus-group exchange described betting as an ordinary intergenerational topic and identified family or religious networks as possible channels of crisis recognition. Source: [FG-S-M, 23], southern focus-group session.	“Betting is an ordinary topic… ‘try it on your club’ is considered normal.” [FG-S-M, 23]	Single-session mechanism illustration; region and gender are source metadata.
Offline peer escalation	One first-person account described face-to-face peer encouragement and rapid stake increases alongside digital payment and re-entry routes. Source: [IG-W-M, 34], western case.	“At the table, they egg you on, ‘come on, chase it.’” [IG-W-M, 34]	Single purposive mechanism illustration; region and gender are source metadata.
Solitary mobile participation and delayed household recognition	One first-person account described concealed phone-based participation and recognition only after debt collection and card blocking. Source: [IG-N-M, 27], northern case.	“I played alone on my phone. No one knew.” [IG-N-M, 27]	Single-case mechanism illustration; region and gender are source metadata.
Acquaintance, influencer, and mentor recruitment	Selected pseudo-investment accounts described recruitment through acquaintances, Instagram, bloggers, mentors, and closed groups. Source: P05 and related selected material; central or eastern metadata retained only where safely reportable.	“I first heard about it among acquaintances… a blogger advertised the project.” [P05]	Selected domain-specific mechanism accounts; region and gender are source metadata.

Table 5 links each contextual mechanism to its evidence source and analytic scope. Table 3, Supplementary material S3 report the case-level audit. Supplementary material S4 reports metadata completeness and contextual-use rules.

A southern focus-group exchange illustrates intergenerational normalization of betting and the role of family or religious networks in crisis recognition. The macro-regional identifier provides source metadata.

“In our context, betting is an ordinary topic. My father and his friends discuss odds over tea. The guys from college are already in the apps; ‘try it on your club’ is considered normal. As long as there are no serious problems, nobody calls it an addiction.” [FG-S-M, 23]

A western first-person account illustrates offline peer escalation through face-to-face encouragement and rapid increases in stakes alongside digital payments and mobile access. The regional identifier provides source metadata.

“In the ‘apartment’ setting, the stakes increase more quickly. You sit at the table, they egg you on, ‘come on, chase it.’ In one evening, I would lose more than in a week online. When I lost <asset redacted>, only then did I realize that I no longer controlled the process.” [IG-W-M, 34]

A northern first-person account illustrates solitary phone-based participation, concealment, and delayed household recognition through debt collection and card blocking. The regional identifier provides source metadata.

“I played alone on my phone. No one knew. My salary went ‘to zero’; I took microloans ‘to cover the debt.’ The family found out when collectors started calling and the bank blocked my card. Before that, it seemed that everything was under control.” [IG-N-M, 27]

Selected pseudo-investment accounts illustrate recruitment through acquaintances, Instagram, Telegram, bloggers, mentors, and closed groups. Central or eastern identifiers are retained as source metadata where safely reportable.

“I first heard about it among acquaintances. Then there were many Instagram reviews saying that people were receiving money, and a blogger advertised the project.” [P05]

Gender markers appear solely in de-identified source identifiers where safely reportable; contextual analysis is organized by mechanism.

### 3.6 Boundary conditions and negative cases across the research questions

Case-level variation included exact, partial, bypassed, recursive, cross-domain, and trial-only sequences. Thirteen cases bypassed at least one intermediate stage, and nine resumed participation in the same domain after crisis/exit. P04 continued into gambling consolidation and escalation after cross-domain entry; P17 stopped after one lottery purchase. No case met the durable-migration criterion.

Offline-first cases placed in-person encouragement, underground venues, and face-to-face pressure within the framework alongside digital payments and mobile re-entry. Family or religious mediation preceded temporary exit in selected accounts. Nine cases documented same-domain re-entry. The cross-domain evidence comprised one continued second-domain case and one trial. These observations define RQ1 through documented entry and retention mechanisms, RQ2 through graded cross-domain evidence, and RQ3 through contextual crisis and response mechanisms.

## 4 Discussion

The findings answer the three research questions through a mechanism-oriented interpretation anchored in case-level variation. Adjacent within-domain links were EXP → ENT in 35 cases, ENT → CON in 39, CON→ESC in 30, and ESC → CRS in 27. Eleven cases followed the exact five-stage within-domain order; 17 were other partial ordered, 12 nonlinear, recursive, or alternative, and 4 trial-only or nonconsolidating. Thirteen unique cases bypassed an intermediate stage. The tested six-element order occurred in zero cases. RQ3 comprises mechanism-centered contextual illustrations interpreted within the documented metadata scope.

The framework reports case-level sequences and cross-case mechanisms at separate evidence levels. The recurrent exposure pattern combined platform visibility, social validation, and entry. Retention accounts described occasional rewards, recovery attempts, debt, rank, and repeated participation. Crisis or exit interrupted participation in some cases, and nine cases documented same-domain re-entry. Participant-level cross-domain evidence consisted of one continued P → G case and one one-off P → G trial after crisis. The strong migration criterion was met in zero cases. Direct records contained zero G → P cases under general elicitation. The framework identifies recurrent stages and empirically uneven transition possibilities.

### 4.1 Interpretation, implications, and future research

Gambling and pyramidal pseudo-investment schemes in Kazakhstan have distinct legal and commercial status and share infrastructures of mobile visibility, social legitimation, payment, credit, and recovery-oriented narration. The analysis states three evidentiary claims. Shared entry and retention mechanisms recur across the two domains. Within-case temporal sequences establish participant-level events with graded evidentiary strength. Cross-source material documents shared cross-domain infrastructure. Mechanism synthesis relies on cross-case evidence, and individual chronology relies on direct first-person evidence.

Previous studies have emphasized persistent availability, Internet-mediated access, gambling marketing, social-media exposure, operator advertising strategies, and digital payment affordances (Gainsbury, 2015; Guillou-Landréat et al., 2021; James and Bradley, 2021; Singer et al., 2024; Lakew et al., 2024). The Kazakhstani cases linked trial entry to platform visibility, instant access, bonuses, or small deposits and to validation by peers, family members, bloggers, mentors, or closed chats. This narrative co-occurrence supports a socially mediated interpretation of platform affordances within the cases examined.

Retention narratives identified domain-specific forms of repeated participation. In gambling accounts, occasional wins and loss chasing sustained further participation. Pseudo-investment accounts linked it to small payouts, credited accruals, package upgrading, rank preservation, referral obligations, and reinvestment. These findings extend Musaraj's (2011) account of pyramids operating through kinship, material, and status relations and complement Ryzhkova and Kashapova’s (2023, 2024) analyses of digital financial pyramids. Debt was reported after harmful participation and during later recovery attempts; prospective evidence is required to estimate an independent causal effect.

Cross-domain participation appeared in two first-person cases. Scholarship on gamblified finance shows that financialized interfaces may incorporate immediacy, reward cues, perceived skill, and high transaction frequency (Andrade and Newall, 2023; Packin et al., 2025; Leong Wei Lyn et al., 2025). Among the 19 pseudo-investment interviews with systematic adjacent-risk elicitation, P04 entered repeated betting after a large win and later losses, and P17 purchased one lottery ticket while expressing a recovery expectation. The records contain no explicit durable substitution or reorientation. Direct gambling records contained zero G → P cases under general elicitation. Law-enforcement and focus-group sources documented overlapping channels, bots, advertising formats, and payment routines as X-level shared-infrastructure evidence.

The contextual material illustrates intergenerational normalization and crisis recognition, offline peer escalation, solitary mobile participation with delayed household recognition, and acquaintance, influencer, or mentor recruitment. Each observation is tied to a selected source. Regional identifiers and gender markers serve as descriptive metadata.

The documented mechanisms identify candidate targets for future intervention evaluation. Entry-stage targets include digital visibility, messenger-based recruitment, registration bonuses, small-deposit prompts, rapid payment affordances, and trusted social validation. Retention-stage targets include loss chasing, perceived control, shame, status, recovery narratives, debt-focused support, and household budgeting. Crisis and exit evaluation can incorporate the nine same-domain re-entry cases, the continued P → G case, and the one-off P → G trial. X-level shared-infrastructure evidence supports product-agnostic monitoring of shared channels; prospective participant-level research can test cross-domain substitution.

Future evaluations can test clearer risk warnings before registration, limits on bonus-based prompts, delayed re-entry after losses, and separation of gambling or pseudo-investment content from ordinary entertainment feeds. Age verification can be assessed at registration, payment, and advertising-delivery points. Where legally and technically feasible, self-exclusion can be examined across operators, payment systems, and related financial channels. Evaluation should measure efficacy, displacement to illegal or mirror channels, privacy and due-process risks, household-debt effects, and movement into adjacent products.

The evidentiary scope follows from purposive, retrospective, multi-source sampling. The purposive sample concentrates accounts involving harm, debt, family crisis, or professional and institutional visibility. Direction-specific P → G reporting uses the 19 pseudo-investment interviews with systematic adjacent-risk elicitation; the G → P count derives from general elicitation in the gambling interviews. Region was reportable for 20 of 44 first-person cases, and each macro-region contributed one focus group. The de-identified archive contains no group-specific distributions of residence, age, gender, recruitment route, language, mode, interviewer, dyad linkage, transcript-level coding, or numerical saturation stopping points. Recall, concealment, shame, and social-desirability pressures may have influenced the accounts. All numerical results represent evidentiary coverage within the purposive corpus.

Population surveys, longitudinal cohorts, ethically available platform or payment metadata, and cross-jurisdictional regulatory studies can extend these findings. Prospective designs can test whether exposure becomes entry, whether crisis/exit is durable, and whether participants move between domains in either direction. Experimental or quasi-experimental designs can evaluate digital access constraints, self-exclusion, debt support, and family or community interventions. Transfer to other jurisdictions requires theory-guided empirical assessment because the mechanisms described here operate within Kazakhstan’s regulatory environment, payment and microcredit infrastructures, digital routines, and social networks.

## 5 Conclusion

Drawing on multi-source qualitative material from Kazakhstan, the study identifies five recurrent within-domain stages and empirically uneven transition possibilities. Eleven of 44 first-person cases documented the exact five-stage order; 17 contained other partial ordered sequences, 12 were nonlinear, recursive, or alternative, and 4 were trial-only or nonconsolidating. Adjacent links were EXP → ENT in 35 cases, ENT → CON in 39, CON→ESC in 30, and ESC → CRS in 27; 13 unique cases bypassed an intermediate stage. The tested six-element order occurred in zero cases. Among the 19 pseudo-investment interviews with systematic adjacent-risk elicitation, two post-crisis P → G entries were documented. One progressed to repeated second-domain participation, and one remained a single trial. The strong migration criterion was met in zero cases. General elicitation in the gambling interviews yielded no G → P case. The contribution is an integrated account of recurrent mechanisms, adjacent within-case transitions, graded participant-level cross-domain evidence, and shared cross-domain infrastructure. Contextual illustrations are interpreted at mechanism level.

## Data Availability

The de-identified analytic dataset supporting the reported case-level counts is provided in Supplementary data S1. Public access and third-party sharing of raw participant data, including audio recordings, full transcripts, source documents, and source-linkage files, are prohibited under the current ethics approval and consent framework because disclosure could compromise confidentiality and create a foreseeable risk of re-identification. Supplementary data S1 contains only public case IDs, de-identified case-level codes, sequence classifications, transition summaries, cross-domain classifications, evidence-provenance rules, metadata-completeness information, and methodological audit displays. It excludes direct identifiers, restricted source-record identifiers, participant-identification keys, demographic linkage keys, and raw narrative materials. Selected de-identified quotations are reported in the article under public case or quotation IDs where required to preserve analytic provenance. Requests may be considered only for additional aggregate or de-identified analytic documentation and remain subject to institutional ethics approval, the scope of participant consent, and confidentiality safeguards.
