# Supplementary material for: Shared digital, social, and cognitive mechanisms in gambling and pseudo-investment schemes in Kazakhstan: a multi-source qualitative study
Source: Front Psychol. 2026 Aug 26;17:1756586. doi: 10.3389/fpsyg.2026.1756586 (PMC13553542; doi:10.3389/fpsyg.2026.1756586)
Supplement: Supplementary file 1 [file Supplementary_file_1.DOCX]

**Supplementary Materials**

**Shared Digital, Social, and Cognitive Mechanisms in Gambling and Pseudo-Investment Schemes in Kazakhstan: A Multi-Source Qualitative Study**

Contents

- S1. Full codebook with operational definitions, evidence provenance, and research-question alignment.
- S2. Abbreviated literature-search protocol and evidence map.
- S3. De-identified case-level sequence matrix, configuration hierarchy, adjacent-transition and bypass counts, graded cross-domain classification, case audit, and evidence-provenance audit.
- S4. Metadata completeness and contextual-illustration audit, including regional missingness, descriptive metadata use, and analytic scope.
- S5. Cross-source integration, discordance handling, group-specific code stability, research-team roles, and recruitment and data-collection audit.
- Supplementary Data S1. Public, de-identified case-level trajectory audit workbook.

Interpretive rule. Supplementary Material S3 and Supplementary Data S1 provide participant-level case, sequence, adjacent-transition, and cross-domain counts. Supplementary Material S4 documents metadata completeness and selected mechanism-centered contextual material. Supplementary Material S5 documents sequential integration of D, C, and X evidence, discordance handling, code stability, author-level responsibilities, and available recruitment and data-collection metadata. S4 and S5 retain their contextual and methodological evidentiary roles.

# Supplementary Material S1. Full codebook with operational definitions, evidence provenance, and research-question alignment

Purpose. The codebook defines five recurrent within-domain stages: exposure, entry, consolidation, escalation, and crisis/exit. It assigns separate categories to same-domain re-entry, temporally ordered cross-domain entry, continued participation in a second domain, and a stronger migration criterion requiring explicit evidence of durable substitution, reorientation, or functional continuation. It also separates participant-level cross-domain evidence from cross-source evidence that channels, payment routes, administrators, or recovery narratives are shared across domains.

Evidence provenance. D denotes temporal ordering documented within one first-person case. C denotes an unlinked family or co-resident collateral account concerning a gambling-related focal person. X denotes cross-case or cross-source mechanism evidence from family, psychologist, law-enforcement, or focus-group materials. Participant-level chronology and counts derive from D evidence. C and X evidence inform corroboration, qualification, challenge, and mechanism scope.

**Identifier conventions.** Public case IDs G01-G25 and P01-P19 are analytic pseudonyms for de-identified first-person cases in Supplementary Material S3. Selected de-identified quotations may carry the corresponding public case ID where case-level analytic provenance is required. Demographic quotation identifiers of the form [TYPE-REGION-GENDER, AGE] retain the convention used in the manuscript and are distinct from the public case IDs. Memo references identify cross-source or negative-case evidence that is not presented as a respondent quotation.

Research-question mapping. S4 supports RQ3 through metadata completeness and source-specific contextual mechanisms concerning recruitment, visibility, household recognition, and crisis response. S5 supports methodological transparency across RQ1-RQ3 through documentation of evidence integration, discordance, code stability, team roles, and data-collection metadata. Regional and gender identifiers remain descriptive source metadata.

Supplementary Table S1. Analytic codebook

| **Code** | **Operational definition** | **Observable indicators** | **Example ID / memo** | **Stage or analytic location** | **Class** | **Provenance** | **RQ** |
| --- | --- | --- | --- | --- | --- | --- | --- |
| EXP_ALG | Platform-mediated or platform-amplified visibility of gambling or pseudo-investment content. | Feeds, stories, advertisements, recommendations, sponsored posts, or unsolicited content. | [FG-S-M, 19]; S3 case matrix | Exposure | Digital | D and X | RQ1 |
| EXP_SOC | Initial social proof that legitimizes risk through acquaintances, peers, family, influencers, mentors, or closed chats. | Recommendation by a trusted person; mentor; club; family or peer normalization. | [FG-C/E-F, 25-34]; P05 | Exposure | Social | D, C, and X | RQ1 |
| ENT_BONUS | Low-commitment entry through bonuses, free bets, symbolic bets, trial accounts, small deposits, or small initial payouts. | Free bet; first deposit; trial account; small contribution; immediate credited gain. | [IG-C/E-M, 24]; S3 case matrix | Entry | Digital / cognitive | D | RQ1 |
| CON_COG | Routinization and perceived control that sustain repeated participation and selective memory of successful episodes. | Repeated participation; perceived strategy; focus on wins or credited accruals. | [PSY-NA-F, 47]; S3 case matrix | Consolidation | Cognitive | D and X | RQ1 |
| RET_STATUS | Status, referral, rank, or club hierarchies that increase attachment and the perceived cost of exit. | Package upgrades; ranks; referral levels; club recognition; obligations to recruits. | P02; P05; [FP-C/E-F, 35] | Consolidation | Social | D, C, and X | RQ1 |
| ESC_CHASE | Loss-recovery or break-even efforts that increase stakes, deposits, contributions, or participation frequency. | Chasing; reinvestment after warnings; increasing stake or contribution; recovery language. | [IG-N-M, 28]; P17 | Escalation | Cognitive | D, C, and X | RQ1 |
| ESC_DEBT | Continuation financed through microloans, consumer credit, borrowing, asset sales, or reinvestment under debt pressure. | Microloans; collectors; household debt; cover debt; consumer credit. | [FM-S-F, 36]; [FM-W-M, 41]; S3 case matrix | Escalation | Social / financial | D, C, and X | RQ1 |
| CRS_FAMILY | Crisis, interruption, or exit initiated through disclosure, family intervention, budget control, religious mediation, or household-level action. | Family council; card control; disclosure; therapy referral; household monitoring. | [IG-C/E-M, 24]; S3 case matrix | Crisis/exit | Social | D and C | RQ3 / boundaries |
| CRS_SELFEX | Technical, legal, clinical, or self-initiated restriction used to interrupt access or participation. | Self-exclusion; self-blocking; access restriction; therapy; formal help-seeking. | [LE-NA-M, 38]; S3 case matrix | Crisis/exit | Digital / institutional | D, C, and X | RQ3 / boundaries |
| REENTRY_SAME | Renewed participation after crisis or exit within the same product domain. | Return after family intervention; repeated scheme entry; reduced or resumed play. | G04, G12, G23-G25; P02, P05, P16, P19 | Post-crisis recursion | Behavioral / social | D | RQ1-RQ3 boundaries |
| XDE_DIRECT | Temporally ordered entry into a second domain within one first-person case. | Pseudo-investment crisis followed by lottery or betting entry; domain direction is explicit. | P04; P17 | Post-crisis cross-domain entry | Behavioral / cognitive | D | RQ2 |
| XDE_CONT | Cross-domain participation that continues beyond a single trial in the second domain. | Repeated second-domain participation, consolidation, or escalation after entry. | P04 | Continued second-domain participation | Behavioral / cognitive | D | RQ2 |
| MIG_STRONG | Explicit durable substitution, reorientation, or functional continuation across domains. | Participant states that one domain replaced, reorganized, or durably continued the other; sustained second-domain trajectory. | Criterion applied to P04 and P17; no case met it | Strong migration criterion | Behavioral / temporal | D | RQ2 |
| PORT_XDOMAIN | Shared use of channels, administrators, advertising formats, payment routes, or recovery narratives documented at cross-source level. | The same Telegram channel, bot, administrator, payment routine, or recovery narrative promotes multiple domains. | Law-enforcement and focus-group cross-source memo | Cross-source mechanism | Digital / social | X | RQ2 |
| PORT_ACCESS | Adapted access or payment arrangements that sustain participation or enable re-entry despite blocking, distrust, or product change. | Mirror sites; replacement accounts; new wallets; crypto; alternative payment routes. | [LE-NA-M, 38] | Re-entry / shared infrastructure | Digital | X unless linked within one case | RQ2 |

Supplementary Table S1. Analytic codebook (continued)

| **Code** | **Operational definition** | **Observable indicators** | **Example ID / memo** | **Stage or analytic location** | **Class** | **Provenance** | **RQ** |
| --- | --- | --- | --- | --- | --- | --- | --- |
| CTX_REGION | Regional metadata tag retained to locate a selected source where safely reportable. | Region attached to an excerpt or source as descriptive metadata. | [FG-S-M, 23]; [IG-W-M, 34]; [IG-N-M, 27] | Descriptive source metadata | Contextual | D, C, and X | Exploratory RQ3 |
| CTX_GENDER | Gender metadata tag retained in de-identified quotation identifiers where safely reportable. | Gender attached to an excerpt; gender remains descriptive metadata and is not modeled. | P05; contextual-use memo | Descriptive source metadata | Contextual | D, C, and X | Exploratory RQ3 |
| LIM_SINGLE | Boundary cases involving nonconsolidation, single-domain participation, stage bypasses, temporary exit, or a one-off cross-domain trial. | Trial-only participation; single-domain path; bypass; temporary interruption; one lottery purchase without continuation. | G08; P09-P11; P17; S3 configuration audit | Boundary condition | Negative case | D, qualified by C/X | RQ1-RQ3 boundaries |

Note. Public case IDs are analytic pseudonyms independent of restricted source-record identifiers. Demographic quotation identifiers remain a separate convention. Participant-level cross-domain counts require D evidence. XDE_DIRECT records second-domain entry, XDE_CONT records continued second-domain participation, and MIG_STRONG records durable substitution or reorientation. X evidence records shared cross-domain infrastructure. Region and gender remain descriptive source metadata.

# Supplementary Material S2. Abbreviated literature-search protocol and evidence map

**Purpose and scope.** This purposive, structured literature review summarizes scholarship relevant to online gambling, gamblified finance, digital pyramidal or MLM-like schemes, and their intersections in support of the empirical qualitative analysis.

**Databases and period.** Searches were conducted in Scopus, Web of Science Core Collection, PsycINFO, eLIBRARY.ru, and Google Scholar for the period 2003-2025.

**Search clusters.** English- and Russian-language query clusters covered: (1) online gambling, betting, online casinos, gambling marketing, social-media advertising, and digital payments; (2) gamblified finance, gamification of trading, cryptocurrency trading, crypto casinos, and problem gambling; (3) financial pyramids, Ponzi schemes, MLM-like schemes, online recruitment, closed chats, and referral hierarchies; and (4) cross-domain terms such as platform affordances, risk environment, cross-domain migration, social proof, loss chasing, and debt escalation.

**Inclusion logic.** Peer-reviewed empirical and review publications were prioritized. For Kazakhstan, legal, policy, statistical, market, economic, and criminological sources were retained when they provided context for digital exposure, advertising, regulation, microfinance, illegal gambling, or market scale.

**Screening summary.** After screening and deduplication, the working corpus comprised 40 sources. Twenty-two source clusters were read in full text or used as core context sources and are listed below. Full bibliographic details are provided in the manuscript reference list.

**Terminology note.** The evidence map writes Digital, Social, and Cognitive in full. The letters D, C, and X are reserved in S1 and S3 for direct, collateral, and cross-source evidence provenance.

**Supplementary Table S2a. Evidence map: core academic literature**

| **Source / source cluster (year)** | **Country/region** | **Method/design** | **Key mechanism or role** | **Brief relevance for the article** |
| --- | --- | --- | --- | --- |
| Gainsbury (2015) | International | Review | Digital; cognitive | Constant online access and internet-mediated gambling are treated as risk-relevant drivers of exposure, repeated participation, and gambling-related harm. |
| Guillou-Landréat et al. (2021) | International | Systematic review | Digital; social | Internet gambling marketing and social-media strategies increase visibility and normalization; supports the need to examine exposure channels. |
| James & Bradley (2021) | International | Systematic review of social-media research | Digital | Social media are a key environment for gambling exposure and for studying gambling-related risk signals. |
| Singer et al. (2024) | International | Systematic review | Digital; social | Operators use advertising strategies on social media; supports analysis of normalized and persistent exposure. |
| Lakew et al. (2024) | Europe | Exploratory focus group / qualitative pilot | Digital | Digital payment solutions may simplify payment and accelerate staking and re-entry. |
| Moravec et al. (2025) | Europe | Cross-sectional study | Digital; social | Social-media engagement is associated with gambling severity; supports cautious linkage between digital visibility and risk. |
| Andrade & Newall (2023) | International | Conceptual review | Digital; cognitive | Cryptocurrencies and “crypto casinos” are conceptualized as gamblified financial risk forms. |
| Packin et al. (2025) | USA / international | Legal / policy analysis | Digital; cognitive | Gamified trading interfaces and transaction-frequency incentives are treated as potential harm mechanisms. |
| Leong Wei Lyn et al. (2025) | International | Narrative review | Cognitive | Stock and cryptocurrency trading and problem gambling share cognitive and behavioral overlaps. |
| Musaraj (2011) | Albania / post-socialist context | Ethnography | Social; cognitive | Pyramids operate through kinship, material, symbolic, and status relations; this supports social legitimation analysis. |
| Ryzhkova & Kashapova (2023) | Russia / CIS | Expert / policy analysis | Digital; social | Digital financial pyramids are analyzed through collective expert assessment; supports platform-mediated pyramid analysis. |
| Ryzhkova & Kashapova (2024) | Russia / CIS | Behaviorally informed policy analysis | Digital; social; cognitive | “Sludges” against Ponzi schemes frame anti-pyramid regulation through behavioral mechanisms. |
| Buribayev et al. (2025) | Kazakhstan | Quantitative modeling | Digital; cognitive | Perceived gambling advertising is associated with propensity to gambling addiction; provides Kazakhstan-specific support for digital and cognitive mechanisms. |
| Buribayev & Khamzina (2025) | Kazakhstan | Legal / policy analysis | Digital; social | Discusses gambling industry revenues and harm-reduction efforts in Kazakhstan. |
| Toleubekova & Osmanova (2025) | Kazakhstan | Legal review | Context | Describes the legislative regulation of the gambling business in Kazakhstan. |
| Dzjazikova & Akhmedzhanova (2024) | Kazakhstan | Criminological analysis | Context | Illegal gambling is examined as a factor increasing criminogenic risk. |
| Mukanov et al. (2025) | Kazakhstan | Market / economic analysis | Context | Maps the gambling market and supports the claim that the phenomenon has substantial scale. |

**Supplementary Table S2b. Evidence map: Kazakhstan-specific contextual sources**

| **Source / source cluster (year)** | **Country/region** | **Method/design** | **Key mechanism or role** | **Brief relevance for the article** |
| --- | --- | --- | --- | --- |
| Bureau of National Statistics (2025a, 2025b) | Kazakhstan | Official statistics | Context / digital | Provides ICT, household internet access, and internet-use context for digital exposure. |
| DataReportal (2024) | Kazakhstan | Digital market report | Context / digital | Provides internet-user and social-media user-identity estimates used to contextualize mobile and platform-mediated exposure. |
| Government of Kazakhstan (2024, 2025) | Kazakhstan | Government plan and enforcement reporting | Context | Documents the 2024-2026 counteraction plan and enforcement actions against gambling advertising and illegal websites. |
| Republic of Kazakhstan legal acts (2007, 2012, 2023, 2024) | Kazakhstan | Legislation | Context | Gambling law, microfinance law, online-platform law, and 2024 gambling amendments define the institutional environment. |
| National Bank of Kazakhstan (2025) | Kazakhstan | Official financial-sector data | Context / social-financial | Microfinance organization data provide context for the debt and credit infrastructure discussed in the qualitative material. |

# Supplementary Material S3. De-identified case-level sequence matrix and evidence-provenance audit

**Unit of analysis.** One row represents one substantive first-person interview. The direct denominator comprises 44 cases: 25 gambling-related cases (G01-G25) and 19 pseudo-investment cases (P01-P19). Counts use unique cases as the unit.

**Denominator reconciliation.** The field report records 25 gambling respondents. One unused blank workbook row was excluded, yielding 25 gambling cases and a combined direct first-person denominator of 44.

Coding rule. Stage status was recorded as present, not documented/not ascertainable, or explicitly denied where the source permitted that distinction. An adjacent transition required consecutive stages in the published within-case sequence. Repeated occurrences remained visible as recursion, and each case contributed at most one count to a given transition. P04 second-domain links, ENT(G)→CON(G) and CON(G)→ESC(G), are reported explicitly and normalized into the overall ENT→CON and CON→ESC totals. Participant chronology and counts derive from D evidence. The pseudo-investment guide provided systematic P→G elicitation; the gambling guide provided general G→P elicitation.

Supplementary Table S3a. De-identified first-person case-level sequence matrix

| **Case ID** | **Documented stage order** | **Configuration** | **Cross-domain classification** | **Post-crisis status / recursion** |
| --- | --- | --- | --- | --- |
| G01 | EXP→ENT→CON→ESC→CRS | Ordered single-domain five-stage | None directly documented | No |
| G02 | ENT→CON→ESC→CRS | Other partial ordered | None directly documented | No |
| G03 | ENT→CON→ESC→CRS | Other partial ordered | None directly documented | No |
| G04 | ENT→CON→ESC→CRS→CON | Nonlinear/recursive/alternative | None directly documented | continued controlled play after crisis |
| G05 | EXP→ENT→CON→ESC→CRS | Ordered single-domain five-stage | None directly documented | No |
| G06 | EXP→ENT→ESC→CON→CRS | Nonlinear/recursive/alternative | None directly documented | Alternative order: escalation at initial entry |
| G07 | ENT→CON→ESC→CRS | Other partial ordered | None directly documented | No |
| G08 | EXP→ENT→CRS | Trial-only/nonconsolidating | None directly documented | No |
| G09 | EXP→ENT→CON | Other partial ordered | None directly documented | No |
| G10 | EXP→ENT→CON→ESC→CRS | Ordered single-domain five-stage | None directly documented | No |
| G11 | ENT→CON→ESC→CRS | Other partial ordered | None directly documented | No |
| G12 | EXP→ENT→CON→ESC→CRS→CON | Nonlinear/recursive/alternative | None directly documented | resumed/reduced play after intervention |
| G13 | ENT→CON→ESC→CRS | Other partial ordered | None directly documented | No |
| G14 | ENT→CON→ESC→CRS | Other partial ordered | None directly documented | No |
| G15 | ENT→CON→ESC→CRS | Other partial ordered | None directly documented | No |
| G16 | EXP→ENT→CON→ESC | Other partial ordered | None directly documented | No |
| G17 | EXP→ENT→CON→ESC→CRS | Ordered single-domain five-stage | None directly documented | No |
| G18 | EXP→ENT→CON→ESC→CRS | Ordered single-domain five-stage | None directly documented | No |
| G19 | EXP→ENT→CON→ESC→CRS | Ordered single-domain five-stage | None directly documented | No |
| G20 | EXP→ENT→CON→ESC | Other partial ordered | None directly documented | No |
| G21 | EXP→ENT→CON | Other partial ordered | None directly documented | No |
| G22 | EXP→ENT→CON→ESC→CRS | Ordered single-domain five-stage | None directly documented | No |
| G23 | ENT→CON→ESC→CRS→CON | Nonlinear/recursive/alternative | None directly documented | continued play after crisis |
| G24 | EXP→ENT→CON→ESC→CRS→CON | Nonlinear/recursive/alternative | None directly documented | continued weekly play after crisis |
| G25 | EXP→ENT→CON→ESC→CRS→CON | Nonlinear/recursive/alternative | None directly documented | continued daily play after family controls |
| P01 | EXP→ENT→CON→CRS | Other partial ordered | None directly documented | No |
| P02 | EXP→ENT→CON→ESC→CRS→ENT | Nonlinear/recursive/alternative | None directly documented | repeated entry into additional schemes |
| P03 | EXP→ENT→CON→ESC→CRS | Ordered single-domain five-stage | None directly documented | No |
| P04 | EXP→ENT→CRS→XDE→ENT(G)→CON(G)→ESC(G) | Nonlinear/recursive/alternative | P→G continued second-domain participation | XDE_DIRECT + XDE_CONT; MIG_STRONG not met |
| P05 | EXP→ENT→CON→CRS→ENT | Nonlinear/recursive/alternative | None directly documented | later same-domain/speculative entry |
| P06 | EXP→ENT→CON→ESC→CRS | Ordered single-domain five-stage | None directly documented | No |
| P07 | EXP→ENT→CON→CRS | Other partial ordered | None directly documented | No |
| P08 | EXP→ENT→CON→CRS | Other partial ordered | None directly documented | No |
| P09 | EXP→ENT→CRS | Trial-only/nonconsolidating | None directly documented | No |
| P10 | EXP→ENT→CRS | Trial-only/nonconsolidating | None directly documented | No |
| P11 | EXP→ENT→CRS | Trial-only/nonconsolidating | None directly documented | No |
| P12 | EXP→ENT→CON→CRS | Other partial ordered | None directly documented | No |
| P13 | EXP→ENT→CON→ESC→CRS | Ordered single-domain five-stage | None directly documented | No |
| P14 | EXP→ENT→CON→ESC→CRS | Ordered single-domain five-stage | None directly documented | No |
| P15 | EXP→ENT→CON→CRS | Other partial ordered | None directly documented | No |
| P16 | EXP→ENT→CON→ESC→CRS→ENT | Nonlinear/recursive/alternative | None directly documented | repeated same-domain entry |
| P17 | EXP→ENT→CON→ESC→CRS→XDE→ENT(G) | Nonlinear/recursive/alternative | P→G one-off cross-domain trial | XDE_DIRECT only; MIG_STRONG not met |
| P18 | EXP→ENT→CON→CRS | Other partial ordered | None directly documented | No |
| P19 | EXP→ENT→CON→ESC→CRS→ENT→CON→ESC→CRS | Nonlinear/recursive/alternative | None directly documented | Repeated same-domain participation across pyramid schemes; no XDE |

Abbreviations. EXP, exposure; ENT, entry; CON, consolidation; ESC, escalation; CRS, crisis/exit; XDE, temporally ordered cross-domain entry. The suffix (G) identifies gambling stages following a pseudo-investment case. XDE records second-domain entry; continued participation and durable migration require XDE_CONT and MIG_STRONG, respectively. “None directly documented” records the evidence present under the applicable interview guide.

**Supplementary Table S3b. Mutually exclusive first-person case configurations**

| **Configuration** | **G** | **P** | **Total** | **Operational interpretation** |
| --- | --- | --- | --- | --- |
| Complete tested six-element order | 0 | 0 | 0 | Tested EXP→ENT→CON→ESC→XDE→CRS specification within one first-person case. |
| Ordered single-domain five-stage | 7 | 4 | 11 | Exact EXP→ENT→CON→ESC→CRS with no recursion or direct domain change. |
| Other partial ordered | 11 | 6 | 17 | Remaining orderable cases after hierarchical assignment; exact five-stage chronology incomplete. |
| Nonlinear/recursive/alternative | 6 | 6 | 12 | Alternative order (1), same-domain re-entry (9), or post-crisis cross-domain entry (2). |
| Trial-only/nonconsolidating | 1 | 3 | 4 | Entry followed by crisis/exit with no documented consolidation or re-entry. |
| Total first-person cases | 25 | 19 | 44 | One focal participant per matrix row; categories are mutually exclusive. |

Classification hierarchy. Cases with an alternative order, same-domain re-entry, or cross-domain entry were assigned to nonlinear/recursive/alternative. Remaining cases with entry followed by crisis/exit and no documented consolidation were assigned to trial-only/nonconsolidating. Exact EXP→ENT→CON→ESC→CRS cases without recursion were assigned to ordered single-domain five-stage. All remaining orderable cases were assigned to other partial ordered. The tested six-element specification occurred in zero cases. P04 met the continued-participation criterion. P17 remained a one-off trial. The strong migration criterion was met in zero cases.

Supplementary Table S3c. Adjacent within-case transition coverage and bypass audit

| **Adjacent transition / audit item** | **G** | **P** | **Total** | **Interpretation** |
| --- | --- | --- | --- | --- |
| EXP→ENT | 16 | 19 | 35 | Exposure and first participation were adjacent in the case sequence. |
| ENT→CON | 23 | 16 | 39 | Entry and consolidation were adjacent; P04 contributes through ENT(G)→CON(G). |
| CON→ESC | 21 | 9 | 30 | Consolidation and escalation were adjacent; P04 contributes through CON(G)→ESC(G). |
| ESC→CRS | 19 | 8 | 27 | Escalation and crisis/exit were adjacent in the case sequence. |
| CON→CRS | 1 | 7 | 8 | Adjacent link; seven cases contained no documented escalation. G06 contained escalation earlier in the episode. |
| ENT→ESC alternative order | 1 | 0 | 1 | G06 escalated during the initial episode before consolidation. |
| ESC→CON alternative order | 1 | 0 | 1 | G06 subsequently consolidated before crisis/exit. |
| ENT→CRS without CON | 1 | 4 | 5 | Entry proceeded directly to crisis/exit without documented consolidation. |
| CON→CRS without documented ESC | 0 | 7 | 7 | Subset of CON→CRS; no escalation was documented in the relevant episode. |
| Any bypass of at least one intermediate stage | 2 | 11 | 13 | Unique cases across ENT→ESC, ENT→CRS without CON, and CON→CRS without documented ESC. |
| CRS→ENT/CON same-domain re-entry | 5 | 4 | 9 | Crisis/exit was followed by renewed participation in the same domain. |
| CRS→XDE, P→G | 0 | 2 | 2 | Pseudo-investment crisis was followed by temporally ordered entry into gambling. |
| XDE→ENT(G) | 0 | 2 | 2 | Both cross-domain sequences reached entry in gambling. |
| ENT(G)→CON(G), continued second-domain participation | 0 | 1 | 1 | P04 continued beyond a trial into repeated gambling. |
| CON(G)→ESC(G), continued second-domain participation | 0 | 1 | 1 | P04 subsequently escalated in gambling. |
| Cross-domain sequence ending after ENT(G) | 0 | 1 | 1 | P17 reported one lottery purchase without documented continuation. |
| Confirmed durable migration | 0 | 0 | 0 | No case documented explicit durable substitution, reorientation, or functional continuation across domains. |
| ESC→XDE | 0 | 0 | 0 | No first-person case supported cross-domain entry before crisis/exit. |
| XDE→CRS | 0 | 0 | 0 | No first-person case supported cross-domain entry followed by crisis/exit in the second domain. |

Note. Transition rows overlap, and each case contributes at most one count to a given link. The CON→CRS without documented ESC row is a subset of CON→CRS. The any-bypass row is a unique-case aggregate across the specified bypass configurations. ENT(G)→CON(G) and CON(G)→ESC(G) are included in the overall ENT→CON and CON→ESC counts. Sequential cross-domain entry, continued second-domain participation, and durable migration are reported as separate evidentiary levels.

**Supplementary Table S3d. Stage coverage in first-person cases**

| **Stage** | **G** | **P** | **Total** | **Interpretive status** |
| --- | --- | --- | --- | --- |
| EXP | 16 | 19 | 35 | Documented in the first-person case; nonmention was not coded as absence. |
| ENT | 25 | 19 | 44 | Documented in the first-person case; nonmention was not coded as absence. |
| CON | 24 | 16 | 40 | Documented in the first-person case; includes P04 consolidation in gambling after XDE. |
| ESC | 22 | 9 | 31 | Documented in the first-person case; includes P04 escalation in gambling after XDE. |
| XDE | 0 | 2 | 2 | Temporally ordered entry into a second domain; durable migration requires the MIG_STRONG criterion. |
| CRS | 21 | 19 | 40 | Documented in the first-person case; nonmention was not coded as absence. |

**Supplementary Table S3e. Evidence provenance by source group**

| **Level** | **Source group** | **N** | **Permitted analytic role** | **Participant-trajectory denominator** |
| --- | --- | --- | --- | --- |
| D | Persons reporting gambling-related problems | 25 | Direct stage presence, adjacent links, bypasses, crisis, and re-entry. | Included |
| D | Pseudo-investment participants | 19 | Direct stage presence, adjacent links, crisis, same-domain re-entry, two P→G entries, one continued second-domain participation case, and one one-off trial. | Included |
| C | Family members or co-residents affected by gambling-related practices | 22 | Separate collateral evidence on harms, debt, concealment, crisis, and household intervention; no public linked-dyad count. | Excluded from direct counts |
| X | Practicing psychologists/psychotherapists | 22 | Cross-case mechanism corroboration, qualification, and negative-case audit. | Excluded |
| X | Law-enforcement officials | 12 | Institutional evidence on shared channels, bots, mirror sites, payment routes, and enforcement barriers. | Excluded |
| X | Macro-regional focus groups | 5 sessions, 6-8 participants each | Community and macro-regional evidence on visibility, normalization, recruitment environments, and coping. | Excluded |

Rule. Participant-level stages, transitions, and counts derive from D evidence. Cross-source convergence supports mechanism interpretation, and discordance narrows claim scope. The public archive reports C evidence at collateral-group level. XDE_DIRECT, XDE_CONT, and MIG_STRONG identify second-domain entry, continued second-domain participation, and durable substitution or reorientation, respectively.

**Supplementary Table S3f. Direction-specific cross-domain and post-crisis audit**

| **Finding** | **Direct case count** | **Evidence level** | **Direction-specific elicitation set** | **Interpretation** |
| --- | --- | --- | --- | --- |
| P→G cross-domain entry after pseudo-investment crisis | 2 (P04, P17) | D | 19 pseudo-investment interviews; later betting, casino, cryptocurrency, or trading activity explicitly elicited. | Two of 19 systematically elicited pseudo-investment cases and two of 44 first-person cases overall. Both entries followed loss or crisis. P04 met XDE_CONT; P17 met XDE_DIRECT. |
| Continued P→G second-domain participation | 1 (P04) | D | Same 19 pseudo-investment interviews. | P04 entered gambling, repeated participation after a large win, and subsequently described further losses. |
| One-off P→G cross-domain trial | 1 (P17) | D | Same 19 pseudo-investment interviews. | P17 bought one lottery ticket under a recovery-oriented script; no repetition, consolidation, or escalation was documented. |
| Confirmed durable migration | 0 | D | All 44 first-person cases assessed under the strong criterion; no case met it. | The strong criterion was unmet in P04 and P17 because the records contain no durable substitution or reorientation. |
| G→P | 0 directly observed | D | 25 gambling interviews; complete pseudo-investment history not systematically elicited. | Zero directly observed under general elicitation across the 25 gambling interviews. |
| G→P shared channels and recovery narratives | No unique case count | X | Law-enforcement and focus-group sources; no participant-level denominator. | Overlapping Telegram, Instagram, TikTok, bot, payment, and promotional infrastructures were reported across domains. |
| Bidirectional cross-domain movement | 0 | D | 44 first-person cases; detection constrained by guide asymmetry. | No first-person case documented movement in both directions. |
| Same-domain re-entry after crisis/exit | 9 | D | 44 first-person cases. | Five gambling cases and four pseudo-investment cases documented renewed participation in the same domain. |
| ESC→XDE and XDE→CRS | 0 and 0 | D | 44 first-person cases assessed through the published within-case sequence. | The tested placement of cross-domain change between escalation and crisis/exit was not supported by a direct first-person case. |

Supplementary Table S3g. Case-level audit of the two cross-domain cases and a same-domain reference case

| **Case ID** | **First-domain sequence/evidence** | **Post-crisis behavior** | **Classification** | **Strong-migration criterion** | **Analytic role** |
| --- | --- | --- | --- | --- | --- |
| P04 | EXP→ENT→CRS in the pseudo-investment domain. | Later lotteries and betting; a large win; repeated gambling; later losses. | XDE_DIRECT + XDE_CONT: continued second-domain participation. | Not met: no explicit durable substitution, reorientation, or functional continuation. | Shows that continued second-domain participation may follow crisis. |
| P17 | EXP→ENT→CON→ESC→CRS in the pseudo-investment domain. | One lottery ticket after the statement “What if I get lucky?”; the record contains no repetition. | XDE_DIRECT only: one-off cross-domain trial. | Not met: no continued second-domain participation or durable reorientation. | Separates sequential participation from continued second-domain participation and migration. |
| P19 | EXP→ENT→CON→ESC→CRS→ENT→CON→ESC→CRS across pyramid schemes. | Repeated entry into additional pseudo-investment schemes after earlier collapse. | Same-domain recursion; no XDE. | Not applicable. | Reference case documenting post-crisis renewal within one domain. |

# Supplementary Data S1. De-identified case-level trajectory audit workbook

The accompanying Excel workbook is the public, de-identified analytic audit supporting case-level counts and method transparency. It contains the 44-row direct case matrix, observed and normalized adjacent-transition listings, configuration and stage summaries, a direction-specific Cross-domain audit sheet, graded cross-domain classifications, provenance rules, source reconciliation, the P04-P17-P19 case audit, metadata-completeness sheets, and sheets for integration, discordance, code stability, team roles, and recruitment mode. Public analytic identifiers and the restricted source-level linkage used for internal validation are maintained in separate records.

## Ethics and confidentiality note

These supplementary materials contain de-identified analytic, aggregate, descriptive, and methodological information. Raw transcripts, audio recordings, source documents, direct identifiers, restricted source-record identifiers, participant-identification keys, and source-level linkage remain in the restricted institutional archive. Selected de-identified quotations may carry a public case ID to preserve analytic provenance.

# Supplementary Material S4. Metadata completeness and contextual-use audit

Unit of analysis. Table S4a uses source groups or focus-group sessions to document safely reportable regional metadata. Table S4b links selected mechanism-centered contextual illustrations to their evidence source and analytic scope. Participant-level sequence and transition counts remain in S3 and Supplementary Data S1. Region and gender function as descriptive source metadata.

Relation to S3, S5, and Supplementary Data S1. Supplementary Material S3 and Supplementary Data S1 provide case-level sequence, adjacent-transition, and cross-domain evidence. S4 records regional metadata for 20 of 44 first-person cases, 24 unavailable or suppressed records, small regional cells, and one focus-group session per macro-region. S5 documents cross-source integration, discordance, code stability, team roles, and data-collection metadata.

Research-question mapping. S4 supports exploratory RQ3 through metadata completeness and source-specific contextual mechanisms. The documented scope is hypothesis-generating and mechanism-centered.

Supplementary Table S4a. Regional metadata completeness and permitted descriptive use

| **Source group** | **N / unit** | **Region safely reportable** | **Region NA or suppressed** | **Permitted analytic use** | **Analytic scope** |
| --- | --- | --- | --- | --- | --- |
| Persons reporting gambling-related problems | 25 | 12 | 13 | Mechanism reconstruction; selected contextual illustration. | Region and gender retained as descriptive source metadata. |
| Pseudo-investment participants | 19 | 8 | 11 | Mechanism reconstruction; selected contextual illustration. | Region and gender retained as descriptive source metadata. |
| Combined first-person trajectory corpus | 44 | 20 | 24 | Participant-level sequence denominator in S3; contextual illustration where metadata are safe. | Contextual mechanism interpretation; region and gender remain descriptive metadata. |
| Family members or co-residents | 22 | 10 | 12 | Collateral context on harm, concealment, and intervention. | Excluded from first-person trajectory counts. |
| Psychologists or psychotherapists | 22 | 11 | 11 | Cross-case mechanism corroboration and qualification. | Professional mechanism interpretation at X level. |
| Law-enforcement officials | 12 | 4 | 8 | Institutional evidence on shared channels, payments, and enforcement barriers. | Institutional and infrastructure interpretation at X level. |
| All individual interview groups | 100 | 45 | 55 | Overall metadata-completeness audit only. | Heterogeneous contextual source groups outside the participant-trajectory denominator. |
| Macro-regional focus groups | 5 sessions | One session in each S/W/N/C/E macro-region | Not applicable | Macro-regional discussion context and selected illustrations. | One session per macro-region; no population, regional-pattern, or gender-group inference. |

Supplementary Table S4b. Selected contextual mechanisms and analytic scope

| **Contextual mechanism** | **Illustrative observation** | **Evidence source** | **Analytic scope** |
| --- | --- | --- | --- |
| Intergenerational normalization and crisis recognition | One focus-group exchange described betting as an ordinary intergenerational topic and identified family or religious networks as possible channels of crisis recognition. | One macro-regional focus-group excerpt [FG-S-M, 23]; southern session metadata. | Single-session mechanism illustration; region and gender are source metadata. |
| Offline peer escalation | One first-person account described face-to-face peer encouragement and rapid stake increases alongside digital payment and re-entry routes. | Selected first-person account [IG-W-M, 34]; western case metadata. | Single purposive mechanism illustration; region and gender are source metadata. |
| Solitary mobile participation and delayed household recognition | One account illustrated concealed phone-based participation and recognition only after debt collection and card blocking. | Single first-person account [IG-N-M, 27]; northern case metadata. | Single-case mechanism illustration; region and gender are source metadata. |
| Acquaintance, influencer, and mentor recruitment | Selected pseudo-investment accounts described recruitment through acquaintances, Instagram, bloggers, mentors, and closed groups. | P05 and related selected material; central or eastern metadata where safely reportable. | Selected domain-specific mechanism accounts; region and gender are source metadata. |

# Supplementary Material S5. Cross-source integration, discordance handling, code stability, research-team roles, and data-collection audit

Purpose. S5 documents respondent-group integration, the inferential roles of D, C, and X evidence, cross-source discordance and intercoder adjudication, group-specific code stability, author-level analytic responsibilities, interviewer protocols, and available recruitment and data-collection metadata. Participant-level counts remain those reported in S3.

Available method metadata. The de-identified archive records the corpus-level double-coded proportion and author-level responsibilities. Participant-family dyad linkage, transcript-level coder assignments, group allocation of the approximately 23% double-coded subset, interview-by-interviewer assignments, route-specific recruitment counts, a complete urban-rural distribution, and source-group distributions by mode, language, age, and gender were unavailable.

**Supplementary Table S5a. Cross-source integration joint display**

| **Model element or claim** | **Direct first-person evidence (D)** | **Collateral/cross-source contribution (C/X)** | **Discordant or limiting evidence** | **Integration decision** |
| --- | --- | --- | --- | --- |
| Exposure to entry | EXP→ENT in 35 first-person cases. | Focus groups and law-enforcement interviews elaborated platform visibility, social proof, and low-barrier access. | Nine gambling-related accounts contained no adjacent EXP→ENT link; these records remained unclassified for that transition. | Retained as a recurrent cross-case link documented in 35 cases; no prevalence estimate assigned. |
| Entry to consolidation | ENT→CON in 39 first-person cases. | Family and psychologist materials elaborated routine formation, concealment, perceived control, and status attachment. | Five cases showed ENT→CRS without documented consolidation; four were classified as trial-only/nonconsolidating. | Retained as a recurrent link documented in 39 cases; five cases proceeded directly from entry to crisis/exit. |
| Consolidation to escalation | CON→ESC in 30 first-person cases. | Family, psychologist, and law-enforcement materials elaborated loss recovery, debt, borrowing, and repeated payment mechanisms. | Eight cases showed CON→CRS; seven had no documented escalation. Thirteen cases bypassed at least one intermediate stage. | Retained as an empirically uneven and conditional transition. |
| Crisis/exit and recursion | ESC→CRS in 27 cases; same-domain re-entry after crisis in 9 cases. | Family accounts elaborated household intervention and concealment; psychologist materials elaborated relapse and delayed help-seeking. | Crisis/exit durability varied; participant-transition counts derive from D evidence. | Crisis/exit retained as a recurrent stage with an explicit recursive pathway. |
| Post-crisis P→G entry | Two P→G entries: P04 continued to consolidation and escalation; P17 remained a one-off trial. | Law-enforcement and focus-group sources documented shared channels and recovery narratives. | Only one case continued in the second domain; no case met MIG_STRONG; guide asymmetry limits direction-specific inference. | Reported as an optional post-crisis event in two cases: one continued case and one trial. |
| Shared cross-domain infrastructure | Shared-infrastructure evidence carries no first-person case count. | X evidence documents overlapping channels, bots, advertising formats, payment routes, and product repackaging. | Documents shared cross-domain infrastructure at X level. | Classified as shared-infrastructure evidence at X level. |
| Contextual recruitment, visibility, and response | Selected D cases illustrate offline peer escalation, solitary mobile participation, and recruitment; no denominator-based inference. | C and X sources elaborate household recognition, intergenerational normalization, and community or institutional context. | Region was reportable for 20 of 44 cases; one focus group represented each macro-region; gender remained descriptive metadata. | Reported as hypothesis-generating, mechanism-centered contextual observations. |

**Supplementary Table S5b. Discordance and adjudication protocol**

| **Issue type** | **Evidence or decision priority** | **Review procedure** | **Reporting outcome** |
| --- | --- | --- | --- |
| Intercoder code assignment or segment boundary | No coder or majority vote had automatic priority. | Reinspect the source-language segment; assess the codebook definition, evidence provenance, and analytic memo; discuss to consensus. | Agreed code retained. Persistent ambiguity coded as not documented/not ascertainable. |
| Temporal order or stage presence | D-level first-person chronology governs participant sequence. | Check explicit temporal markers, episode boundaries, and adjacency; count consecutive stages as adjacent transitions. | Unresolved order remains uncounted; no stage is inferred from nonmention. |
| First-person and family/co-resident interpretations | D governs participant chronology; C concerns observed harm, concealment, crisis recognition, and household intervention. | Retain both source positions; D governs chronology, and C qualifies contextual interpretation. | Source discordance qualifies visibility or mechanism scope without changing the participant sequence count. |
| Participant evidence and professional, institutional, or community generalization | D governs participant-level events and counts; X governs cross-case, institutional, or contextual interpretation. | Audit X claims against the direct-case matrix and negative cases. | X corroborates or narrows mechanism scope at cross-source level. |
| Shared infrastructure and migration classification | MIG_STRONG requires explicit D-level durable substitution, reorientation, or functional continuation. | Separate shared channels and recovery narratives from XDE_DIRECT, XDE_CONT, and MIG_STRONG. | Shared-infrastructure evidence retained at X level; durable migration count remains zero. |
| Translation disagreement | Source-language meaning has priority over literal English form. | Forward translation by one bilingual researcher; review by a second bilingual researcher; review semantic accuracy, pragmatic force, and context; record memo when needed. | Consensus English excerpt prioritizes conceptual equivalence. |
| Missing, conflicting, or unsafe metadata | No imputation from quotation context or external knowledge. | Check the de-identified source record and confidentiality rule. | Record NA, insufficiently specific, or suppressed; no subgroup inference. |

**Supplementary Table S5c. Group-specific code-stability and analytic-sufficiency audit**

| **Source group** | **N or unit** | **Primary analytic purpose** | **Separate assessment** | **Reporting conclusion** |
| --- | --- | --- | --- | --- |
| Persons reporting gambling-related problems | 25 interviews | Direct stages, adjacent transitions, bypasses, crisis, re-entry, and gambling-specific mechanism variation. | Reviewed separately for recurrence of first-cycle codes, stability of code definitions, and negative or alternative cases. | Group-specific code stability supports case mapping within the purposive gambling group. |
| Pseudo-investment participants | 19 interviews | Direct stages, adjacent transitions, scheme-specific mechanisms, and direction-specific elicitation of later adjacent-risk activity. | Reviewed separately for recurrence, late code changes, negative cases, and the final cross-domain prompt. | Group-specific code stability supports direct case mapping and the 19-case P→G elicitation set. |
| Family members or co-residents | 22 interviews | Collateral material on debt, concealment, harm recognition, household intervention, and non-durable exit. | Reviewed as a separate C source group; not used to complete D trajectories. | Analytic sufficiency assessed for collateral mechanisms at source-group level. |
| Psychologists or psychotherapists | 22 interviews | Cross-case elaboration of perceived control, loss chasing, debt distress, family involvement, help-seeking, and relapse risk. | Reviewed separately for mechanism elaboration, qualification, and limiting cases. | No clinical prevalence or professional-population saturation claim. |
| Law-enforcement officials | 12 interviews | Institutional evidence on illegal gambling, fraud infrastructure, channels, mirror sites, payments, repackaging, and enforcement barriers. | Reviewed separately for institutional recurrence and divergence from participant-level evidence. | No participant-behavior or population saturation claim. |
| Macro-regional focus groups | 5 sessions, 6-8 participants each | Contextual material on visibility, normalization, consequences, help-seeking barriers, and coping. | One session per macro-region was reviewed for contextual coverage and cross-session recurrence. | Contextual code stability assessed at session level across one focus group per macro-region. |
| Cross-source synthesis | All source groups after within-group coding | Synthesis of convergence, complementarity, qualification, discordance, and absence by RQ and model element. | Joint display applied only after separate within-group review of code recurrence and stability and construction of the 44-case D matrix. | Source-group code stability preceded cross-source synthesis of convergence, complementarity, qualification, discordance, and absence. |

**Supplementary Table S5d. Research-team and coding-responsibility matrix**

| **Role or record** | **Documented responsibility** | **Validation or decision procedure** | **Reporting scope** |
| --- | --- | --- | --- |
| Z.K. (first author) | Investigation; recruitment and fieldwork supervision; source-level validation; manuscript drafting and review. | Supervised use of the approved semi-structured guides, informed-consent procedures, distress-sensitive interviewing protocol, and confidentiality requirements; validated source-level interpretations. | Author-level fieldwork supervision is documented; interview-by-interviewer assignments were unavailable. |
| Y.B. (corresponding author) | Codebook-oriented analytic structure, formal analysis, case mapping, construction of audit displays, visualization, manuscript drafting and review. | Led analytic construction and participated in consensus review of double-coded discrepancies, disputed interpretations, negative cases, and framework boundaries. | Public reporting is at author-role level; transcript-level coder assignments are not preserved. |
| Two-author consensus process | Joint review of discrepancies arising from the double-coded subset, disputed interpretations, cross-source discordance, negative cases, and the final framework. | Source-language reinspection, codebook and provenance check, memo review, and consensus. | Unresolved ambiguity remained not documented/not ascertainable. |
| Double-coded subset | Approximately 23% of the 100 individual interviews across respondent groups. | Discrepancies reviewed through consensus-calibration; no formal agreement coefficient calculated. | Corpus-level double-coding proportion and consensus procedures documented; transcript and group allocation unavailable. |
| First-person case-map validation | All 44 D-level case maps. | Checked against source-language records for denominator, stages, order, adjacent links, bypasses, recursion, and provenance. | Ambiguous elements were not inferred. |
| Translation review | One bilingual researcher prepared the forward translation and a second bilingual researcher checked it against the source excerpt. | Semantic accuracy, pragmatic force, contextual equivalence, and culturally specific expressions were reviewed. | Only selected publication excerpts were translated; source-language coding was retained. |

**Supplementary Table S5e. Recruitment, participant-characteristic, and data-collection audit**

| **Source group** | **N** | **Recruitment description** | **Mode, language, and duration** | **Reportable characteristics and metadata** | **Available reporting scope** |
| --- | --- | --- | --- | --- | --- |
| Persons reporting gambling-related problems | 25 | Mixed anonymous social-media or messenger announcements, professional/civil-society contacts, and snowball referrals. | Online or in person; Russian and/or Kazakh; usually 50-90 minutes. | Adults; selected urban residence records; region 12 reportable and 13 NA/suppressed. | Region reportable as specified; other group-level distributions unavailable. |
| Pseudo-investment participants | 19 | Mixed announcement, professional/civil-society, and referral channels; actual contribution and approximate participation period required. | Online or in person; Russian and/or Kazakh; usually 50-90 minutes. | Adults; region 8 reportable and 11 NA/suppressed. | Region reportable as specified; other group-level distributions unavailable. |
| Family members or co-residents | 22 | Mixed community, professional/civil-society, and referral channels; household, budget, or sustained-care relationship required. | Online or in person; Russian and/or Kazakh; usually 50-90 minutes. | Adults; region 10 reportable and 12 NA/suppressed. | Collateral-group reporting; other group-level distributions unavailable. |
| Psychologists or psychotherapists | 22 | Professional contacts; direct relevant counselling, clinical, rehabilitation, crisis-support, university-service, or private-practice experience. | Online or in person; Russian and/or Kazakh; usually 50-90 minutes. | Professional setting, approximate experience, and case types where available; region 11 reportable and 11 NA/suppressed. | Functional professional sample; other group-level distributions unavailable. |
| Law-enforcement officials | 12 | Formal institutional channels; functional relevance to fraud, illegal gambling, cybercrime, economic investigation, or enforcement. | Online or in person; Russian and/or Kazakh; usually 50-90 minutes. | Functional roles only; region 4 reportable and 8 NA/suppressed. | Ranks, offices, and route/mode/language/interviewer distributions not reported. |
| Macro-regional focus groups | 5 sessions; 6-8 participants each | Mixed adult community recruitment under the study announcement and referral strategy; route recorded at group level. | Online; Russian and/or Kazakh; approximately 80-90 minutes; one session per macro-region. | Session-level composition; one southern, western, northern, central, and eastern session. | No individual-participant denominator, source-group age/gender distribution, or regional saturation claim. |

Interpretive rule. The method-transparency tables document analysis and reporting of the existing corpus. Missing metadata remain unclassified. The 44 first-person cases retain the sole participant-trajectory denominator. Regional, urban-rural, gender, language, interviewer, and interview-mode variables remain descriptive metadata.
